# Stigma, HIV and health: a qualitative synthesis

**DOI:** 10.1186/s12889-015-2197-0

**Published:** 2015-09-03

**Authors:** Lori A. Chambers, Sergio Rueda, D. Nico Baker, Michael G. Wilson, Rachel Deutsch, Elmira Raeifar, Sean B. Rourke, The Stigma Review Team

**Affiliations:** School of Social Work, McMaster University, Kenneth Taylor Hall, KTH-319, 1280 Main St. West, Hamilton, ON L8S 4M4 Canada; Social and Epidemiological Research Department, Centre for Addiction and Mental Health, c/o Research Services Office, 33 Russell St., T100, Toronto, ON M5S 2S1 Canada; Department of Psychiatry, University of Toronto, 250 College St., 8th floor, Toronto, ON M5T 1R8 Canada; Institute for Work & Health, 481 University Ave., Suite 800, Toronto, ON M5G 2E9 Canada; Ontario HIV Treatment Network, 1300 Yonge St., Suite 600, Toronto, ON M4T 1X3 Canada; Department of Clinical Epidemiology and Biostatistics, McMaster University, Communications Research Laboratory, CRL-209, 1280 Main St. West, Hamilton, ON L8S 4M4 Canada; Centre for Health Economics and Policy Analysis, McMaster University, 1280 Main St. West, Hamilton, ON L8S 4M4 Canada; McMaster Health Forum, McMaster University, Mills Memorial Library, MML-417, 1280 Main St. West, Hamilton, ON L8S 4M4 Canada; Department of Psychiatry, Weill Cornell Medical College, 21 Bloomingdale Rd., White Plains, NY 10605 USA; Centre for Research on Inner City Health, The Keenan Research Centre, Li Ka Shing Knowledge Institute, St. Michael’s Hospital, 209 Victoria St, Toronto, ON M5B 1 T8 Canada

## Abstract

**Background:**

HIV-related stigma continues to negatively impact the health and well-being of people living with HIV, with deleterious effects on their care, treatment and quality of life. A growing body of qualitative research has documented the relationship between HIV-related stigma and health. This review aims to synthesize qualitative evidence that explored the intersections of stigma and health for people with HIV.

**Methods:**

A thematic summary was conducted that was guided by the qualitative metasummary technique developed by Sandelowski and Barraso. Literature searches yielded 8,622 references of which 55 qualitative studies were identified that illustrated HIV-related stigma in the context of health.

**Results:**

The metasummary classified qualitative findings into three overarching categories: conceptualizing stigma which identified key dimensions of HIV-related stigma; experiencing stigma which highlighted experiences of stigma in the health context, and managing stigma which described ways in which stigma is avoided or addressed. To better illustrate these connections, the qualitative literature was summarized into the following themes: *stigma within health care settings*, *the role of stigma in caring for one’s health*, and *strategies to address HIV-related stigma in the health context*. A number of health care practices were identified – some rooted in institutional practices, others shaped by personal perceptions held by practitioners – that could be stigmatizing or discriminatory towards people with HIV. There existed interconnections between enacted stigma and felt stigma that influenced health care utilization, treatment adherence, and overall health and well-being of people with HIV. Intersectional stigma also emerged as instrumental in the stigma experiences of people living with HIV. A number of strategies to address stigma were identified including social support, education, self-efficacy, resilience activities, and advocacy.

**Conclusion:**

This review of the qualitative evidence indicates that HIV-related stigma within health contexts is a broad social phenomenon that manifests within multiple social spheres, including health care environments. Findings from this review indicate that future stigma research should consider the social structures and societal practices – within and outside of health care environments – that perpetuate and reinforce stigma and discrimination towards people with HIV.

**Electronic supplementary material:**

The online version of this article (doi:10.1186/s12889-015-2197-0) contains supplementary material, which is available to authorized users.

## Background

In the early years of the HIV/AIDS epidemic, the social consequences of stigma and discrimination towards people with HIV were identified as part of the “third phase of the epidemic” and addressing these consequences was as “central to the global AIDS challenge as the disease itself” [1]. With the introduction of combination antiretroviral treatments (cART) in 1996, there was optimism that HIV/AIDS and the resulting stigma and discrimination could be addressed. Despite advancements in treatment and the evolution of care to combat HIV/AIDS worldwide, in addition to the proliferation of HIV-related education, work remains to combat the stigma associated with HIV infection. Due to the perceptions and judgements of HIV that continue to persist, stigma remains one of the biggest challenges in the social response to HIV/AIDS.

The concepts of stigma applied in HIV research have been shaped by the seminal work of Erving Goffman (1963) who defined stigma as a discrediting “mark” or attribute which reduces the status of the person in the eyes of society [2]. Though health-related stigma has been associated with other health conditions such as mental illness, cancer, and other sexually transmitted infections, much of the recent illness stigma literature has been devoted to the stigma and discrimination associated with HIV and AIDS (HIV-related stigma) and the development of conceptual frameworks specific to HIV-related stigma [3–9]. These conceptual frameworks have identified particularities of stigma in the context of HIV including: the moral valuations ascribed to the illness, modes of transmission, and populations impacted; the intersections of HIV-related stigma with other forms of social marginalization; and the recognition of societal power relations within mechanisms of stigma [3–9].

There has been a growing body of literature exploring stigma and health for people with HIV. This literature indicates that stigma continues to have a detrimental impact on the health and well-being of people with HIV, and their access to health and social services [10, 11]. Studies have reported on discrimination in healthcare environments towards people with HIV manifesting as denial of care, confidentiality breaches, negative attitudes, and humiliating practices by health care workers [12–14]. Other studies have identified the impact stigma can have on a person’s self-concept or mental health once his or her status is known [15, 16]. Stigma has been shown to impact mental health factors for people with HIV including anxiety [17–19], depression [20–22], suicidal ideation [23, 24], emotional health [25], psychological well being [26], life satisfaction [27], and quality of life [28, 29]. Stigma has also been linked to health care seeking and adherence to antiretroviral medication [30–33]. Consequently, stigma related to HIV remains a daunting barrier to HIV prevention [34]. Fears of disclosure, anticipated stigma, internalized shame, and experiences of discrimination within health care settings and in greater society can influence future decision making around prevention activities [35–38].

Although there has been a number of recent reviews that examined stigma and HIV [16, 39–41, 42], there has been limited research that systematically integrates *qualitative* evidence in its exploration of stigma, HIV and health. Qualitative synthesis, a method of aggregating qualitative evidence, is a burgeoning approach to synthesizing research evidence and has grown in popularity within health research [43–47]. While syntheses of quantitative evidence have been used to understand causal mechanisms, to measure effect size, or to determine intervention effectiveness, qualitative syntheses are better suited for understanding the nature of a phenomenon, to explore contextual features of experience and to develop theoretical concepts derived from findings across studies [48–50]. A synthesis of the qualitative literature on HIV, stigma and health is particularly important given the evolution of the epidemic, its global nature and the diversity of cultures and populations that have been impacted by HIV/AIDS. Qualitative evidence is particularly helpful in understanding the socially constructed nature of HIV infection, interpreting the social processes, interactions, or contextual features that influence health care decision making, and delineating the interconnections between stigma-related processes and related health impacts.

The original objectives of this project were to conduct a synthesis of the qualitative and quantitative research evidence to better understand the impacts of stigma on the health of people with HIV. This paper reports on the qualitative evidence that explored the intersections of stigma and health for people with HIV. In the context of this review, HIV-related stigma is defined as the prejudicial feelings, stereotypical perceptions, discriminatory behaviors and actions, or social devaluation of HIV infection, HIV/AIDS related illnesses, the activities associated with HIV-infection, and people with HIV [2, 6, 51].

## Methods

### Search strategy

From February 2009 to May 2010, a series of electronic and manual searches were carried out to yield qualitative and quantitative literature related to HIV, stigma and health. A librarian conducted a search of six electronic database (CINAHL, EMBASE, Medline, PsycINFO, Sociological Abstracts, and WHOLIS) using keyword terms related to HIV/AIDS (e.g., human immunodeficiency, acquired immunodeficiency syndrome), and stigma/discrimination (e.g., stigma*, ostracism, prejudice, stereotyping, discrimination).

To ensure that important evidence not captured in an electronic database search was not missed, manual search strategies were also performed including: citation searches of systematic reviews addressing the topic of stigma for people with HIV [16, 39, 40, 41, 42, 52], a bibliographical review of articles included after full paper review, and recommendations from content experts knowledgable in HIV-related stigma. The date range of the literature search spanned from January 1996 to 2010. The literature search was restricted to post-1995, since the introduction of cART is often cited as a key turning point in the HIV epidemic. To avoid the exclusion of relevant health-related literature at this stage, the search strategy used key terms for HIV and stigma exclusively, and then incorporated health-related inclusion criteria in the review stages (see Additional file 1 – Screening process for qualitative sysnthesis).

### Reviewing

The inclusion process went through three review stages: title and abstract review to determine clear inclusion, full-paper review to determine general applicability to the synthesis aims, and methodological review to determine applicability based on the pre-specified inclusion criteria. The inclusion criteria were as follows: 1) qualitative studies; 2) published after 1996; 3) that reported thematic findings on stigma and health; 4) and had participants with *experiential knowledge* of stigma based on their own experience (people with HIV) or from the perspective of living with or working with people with HIV (i.e., clinicians, community workers, caregivers, family members of people with HIV). Although language restrictions were not applied as an inclusion criterion, this review was only able to synthesize studies that were written in English, French or Spanish.

### Qualitative analysis

Prior to extracting the data, the authors developed an analytical focus guided by the following questions:

- How is stigma defined in the qualitative literature exploring stigma, health and HIV?
- What are the health-related experiences of stigma for people with HIV?
- What are the ways people with HIV navigate health-related experiences of stigma?

The working definition of health used for this synthesis derived from the health domains (physical health, mental health and substance use, health care access/utilization, and adherence to antiretroviral medications) identified in a parallel quantitative systematic review on the effects of HIV-related stigma on health outcomes that was conducted by some of the authors [53]. The analysis of qualitative literature also accounted for any other health-related findings that may further delineate the interconnection between stigma and health (i.e., achieving wellness while experiencing stigma, secondary health-related factors that may act as a pathway between stigma and health). The conceptualization of stigma used for analysis was shaped by broad conceptual frameworks of stigma such as those proposed by Goffman [2]; Alonzo and Reynolds [3]; and Link and Phelan [54]; and HIV-specific frameworks of stigma such as the work of Herek et al. [8, 9]; and Parker and Aggleton [4]. The authors chose these frameworks as they captured the particularities of stigma in the context of HIV and delineated the social mechanisms that could potentially contribute to emotions, beliefs and behaviours that shape health-related experiences for people with HIV.

A thematic summary guided by the qualitative metasummary technique developed by Sandelowski and Barraso was completed. Qualitative metasummary is a synthesis method used to aggregate qualitative findings and to create a thematic taxonomy across studies [46, 55]. Thematic analytical approaches were also used to identify, interpret, and report overarching themes and patterns of meanings emerging from the included literature [50, 56, 57]. An inductive analytical approach was used so that the themes identified for the synthesis were strongly linked to the thematic findings reported within each study [58].

During the data extraction process, authors’ statements of findings were isolated including quotes specifically related to those findings. Next, similar findings were grouped using a coding key developed from the analysis. The coding key was established using the themes identified in the original studies. Each reviewer coded each study independently, then met to compare coding to ensure consensus. Using constant comparison methods, codes were grouped into themes, then findings were organized thematically based on replication (confirming what is said in other studies), extension (providing additional contextual information that extends findings) or refutation (providing a contrary view to what is said in other studies). Conceptual frameworks for stigma also guided the integration of findings into the overarching themes.

Three reviewers were involved in the data extraction and analysis of qualitative findings (LAC, DNB, and RD), and a fourth reviewer (ER) was involved in the verification of data extraction and thematic coding. Each included reference was reviewed and appraised in duplicate by two independent reviewers. Data extraction was conducted by one reviewer and verified by a second reviewer. Any disagreements between reviewers were resolved by consensus and, if that failed, a third independent reviewer resolved the disagreement. Thematic analysis was conducted by a team of two reviewers with audits conducted by a third reviewer to ensure authenticity of extracted data to the original study, and to ensure consistency of thematic analysis. The qualitative team met regularly to discuss review findings. As part of the peer review process, members of the team for the quantitative review also joined the qualitative review meetings at three review stages: completion of data extraction, completion of thematic coding, and preliminary analysis of qualitative findings.

## Results

### Identification, screening and eligibility

**Fig. 1:**
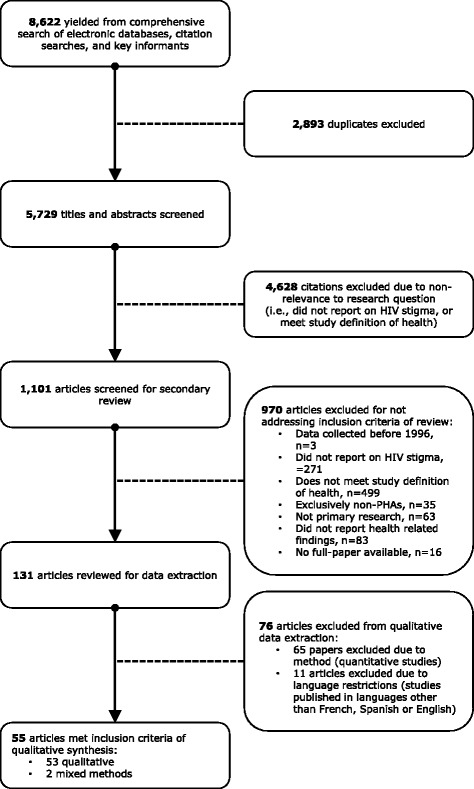
Screening process for qualitative synthesis. Figure 1 illustrates the screening and review process for the qualitative synthesis, number of references/papers excluded at each review stage, and reasons for exclusion

The literature searches yielded 8,622 references (5,729 after duplicate references were removed) of which 131 papers were included for qualitative or quantitative data extraction. From the 131 studies that met the criteria for inclusion, 76 were excluded from the qualitative synthesis (see Additional file 2 for a listing of articles excluded from qualitative data extraction): 65 studies that reported only quantitative findings and 11 studies published in languages other than English, French or Spanish. ^1^ In total, 55 studies were included in the qualitative synthesis: 53 qualitative studies, and two mixed method studies [35, 59–112]. The inclusion process has been documented in Fig. 1.

### Study characteristics

The settings for included studies were geographically diverse, with a mixture of studies conducted in high-, middle- and low-income countries (see Additional file 3 for a summary of study characteristics). Twenty-nine studies were conducted in the Americas (Canada (*n* = 3), Mexico (*n* = 2), Grenada, and  Trinidad and Tobago (*n* = 1), and the United States (*n* = 23)), 10 studies in South-East Asia (Cambodia (*n* = 1), China (*n* = 4), and Vietnam (*n* = 5)), eight in Africa (Botswana (*n* = 1), Ghana (*n* = 1), South Africa (*n* = 2), Uganda (*n* = 2), Zambia (*n* = 2)), four in Western Europe (Ireland (*n* = 1), and the United Kingdom (*n* = 3)), three in South Asia (India), and one in Europe/Central Asia (Russian Federation). Twenty-seven studies focused on a specific population, including women (*n* = 11), children and youth (*n* = 6), ethnoracial communities in Western countries (*n* = 5), people who use drugs (*n* = 2), transgender persons (*n* = 1), people with incarceration histories (*n* = 1), and older people with HIV (*n* = 1). Of the 55 studies included in this qualitative synthesis, only two specifically aimed to study the intersections of HIV, stigma and health [108, 112]. The remaining 53 papers indirectly reported on the intersection of health and stigma within their thematic results: 16 studies explored stigma or discrimination and 37 explored health-related topics. Of the studies where health was the primary aim of the paper, 15 studies focused on adherence [63, 64, 72, 75, 84, 83, 89, 93, 95, 97, 98, 100, 101, 104, 111], 16 on health care access and utilization [35, 59, 62, 73, 80, 81, 85-87, 91, 92, 94, 96, 103, 106, 109], three studies on general quality of life [66, 69, 78], two studies on substance use [71, 110], and one study on mental health [70]. Nineteen studies applied an explicit concept of stigma to their study findings. Goffman’s conception of stigma [2] was most cited; other concepts of stigma and discrimination applied in the qualitative findings include the UNAIDS’ Protocol for the Identification of Discrimination against People with HIV [113]; Link and Phelan’s conceptualization of stigma [54]; Parker and Aggleton’s conceptualization of HIV/AIDS related stigma and discrimination [4], and Alonzo and Reynolds’ definition of stigma [3]. Of these nineteen studies, four developed conceptual stigma frameworks using the original study findings [77, 104, 107, 110].

### Synthesis of qualitative literature

While the qualitative studies on HIV-related stigma were geographically and topically diverse, review findings suggest that the included studies shared some common conceptions of stigma within the health context. In this synthesis, we initially categorized the qualitative findings using the themes commonly identified across the included literature; then, in the thematic analysis, we identified overarching themes in relation to HIV, stigma and health. The metasummary classified qualitative findings into three overarching themes: *conceptualizing stigma *which identified key dimensions of HIV-related stigma; *experiencing stigma* which highlighted experiences of stigma in the health context, and *managing stigma* which described ways in which stigma was circumvented, buffered or combated. (See Additional file 4, for a matrix summary classifying the common themes identified in the included literature; see Additional file 5, for a detailed description of these themes).

The reviewed studies identified six dimensions of HIV-related stigma: *enacted stigma* (discriminatory behaviours, actions or attitudes from others), *felt stigma* (internalization of stigma; devaluing beliefs, behaviours or actions in which people with HIV may hold or engage), *marginalization* (other forms of social devaluation), *disclosure *(disclosure of seropositive status), *morals and values* (social mores and societal values), and *visible health* (visible symptoms of seropositivity). These dimensions informed how stigma was experienced by people with HIV and addressed within health care contexts. The qualitative literature illustrated stigma as a social process, and study findings delineated the ways in which the dimensions of HIV-related stigma manifested within health care access and utilization, adherence to treatments, and maintenance of one’s health and well-being. Lastly, these studies offered various strategies through which stigma was managed. This synthesis of the qualitative literature indicates that the six dimensions of HIV-related stigma are interconnected thereby illustrating the complex dynamics between marginalization, disclosure, social mores and values, and visible health in enacted and felt stigma.

To better illustrate these connections, summarized here are aspects of HIV, stigma and health that were prominent in the qualitative literature. The first section, *HIV and stigma in health care environments* describes some of the ways in which health care practices can be discriminatory towards or interpreted as stigmatizing by people with HIV. The second section, *HIV and stigma and its role in caring for one’s health*, identifies some of the processes and practices people with HIV may use to navigate stigma and discrimination while caring for their health and well-being. In the last section, *strategies identified to address HIV-related stigma* are explored. These modes of addressing stigma offer people with HIV, as well as health care practitioners and other advocates in their care, alternative ways of managing stigma that could contribute to stigma reduction.

### HIV and stigma in health care environments

The literature identified a number of strategies used within health care settings to manage HIV/AIDS. Some of these practices were rooted in prevention strategies, others seemed shaped by personal perceptions that health practitioners held towards HIV/AIDS or people with HIV. Three practice strategies emerged from the literature: risk management, where strategies to mitigate perceived health risks were institutionalized in care; fear management, where care practices stemmed from practitioner fears of HIV exposure; and moral management, where care practices derived from judgmental perceptions towards people with HIV and transmission activities. The literature also identified intersectional stigma, or interconnections between HIV-related stigma and other forms of social marginalization as they inform access to health care. Whether these practices were codified in institutional systems or engaged indiscriminately by health care providers, they were distinguished as stigmatizing health care practices as they had the potential to expose people with HIV to discrimination or enacted stigma.

#### Risk management

A number of risk management procedures institutionalized in organizational policies were characterized as being potentially discriminatory towards people with HIV. Segregation of people with HIV was an example of a precautionary measure that could incite stigma. Some studies considered segregation *with one’s own* [2] – others who live with HIV – a means of coping with the condition, particularly for people who had been recently diagnosed. Other studies noted acts of segregation that may intially have been instituted to protect people with HIV, such as protecting them from opportunistic infections. The qualitative literature also discussed segregation practices as intentionally used to differentiate people with HIV from the general care population. Brickley’s (2007) study remarked that segregation practices within health care settings served as a means of stigmatizing people with HIV [35]:

*“You know, at [the] hospital, when we come, everybody knows who we are. Infected people want as few people to know about their situation as possible. With other diseases like hepatitis, the patients may die sooner but aren’t as discriminated. This disease is repellent. Therefore at [the] hospital, there is a room reserved for the patients of this [HIV/AIDS] disease. Anyone coming there is infected. When we’re waiting outside the room, we will be identified as infected. That room is for this disease. You go elsewhere for other diseases or for consultation. That room is for this disease.” (22- year-old post-partum woman quoted in Brickley (2007), p. 107)*

As illustrated above, segregation as an institutional practice could demark people with HIV. Furthermore, by separating people with HIV from the general public, health institutions could inadvertently disclose serostatus.

Confidentiality violations were another form of disclosure within health care settings discussed as inciting stigma. Institutional practices such as labeling client records or lab work – infection control marking, warning labels on blood or urine tests, or labels on medications – could potentially disclose one’s status. These care practices could subsequently violate patient’s confidentiality, particularly in places where these markings are publically displayed [74]:

*“So when I got there [to the medical clinic] they hand me my chart and I’d go see the blood pressure nurse, then I’d go see the nurse that draws blood, then I’d go see the doctor. I’d walk around with this big chart with this big sticker on it—HIV positive. Everyplace I went I was carrying this. I felt why don’t they just tattoo my forehead.” (“Louis” quoted in Emlet (2007), p. 747)*

Whether confidentiality was violated consciously or unconsciously, enacted by individuals or codified in health care practices, it had a profound impact on how people with HIV experienced health care and on their future health care seeking behaviours.

The qualitative literature identified ways in which health care strategies designed to mitigate health risks could become discriminatory care practices. However, Li et al. (2009) cautions on distinguishing between stigmatizing behaviors and discriminatory intent; stigmatizing behaviors can be determined by a combination of factors such as personal attitudes, social norms, and situational cues. Additionally, historical experiences of discrimination may influence the interpretation of behaviors as discriminatory [114]. Nevertheless, the literature illustrated ways in which institutional practices became stigmatizing forces for a person with HIV even if discrimination was not intended. Behaviors that may seem benign to a health care practitioner may be stigmatizing for a person with HIV, particularly if these actions mirror prior discriminatory experiences within health care settings or are reflective of HIV-related stigma within greater society. Although these institutional risk management strategies could have been considered ethical care protocol without malfeasance, synthesis findings suggest that risk management strategies that declared one’s status through segregation or demarked one’s status through labeling could become forms of disclosure that potentially exposed people with HIV to stigma.

#### Fear management

The qualitative literature also illustrated that misperceptions of HIV transmission could fuel fears of infection. Though these fears were typically described as manifesting in individuals with limited knowledge of HIV transmission, they were also expressed by health care professionals expected to be more knowledgeable about modes of transmission. For example, Rintamaki et al. (2007) indicated that HIV-positive participants often attributed expressions of nervousness among health care staff to fears of transmission [96]:

*“The dentist that was actually going to work on me, I felt like, the vibe that I got from him, the energy that I got from him, or at least, the demeanor that I got from him, was that he really didn’t want to work on me or he wasn’t comfortable working on me. And that was real disconcerting because I thought, “Where am I going to go to be able to get this done?” (“Jerome” quoted in Rintamaki (2007), p. 961)*

Other examples of fear management were excessive precautions used by practitioners such as wearing protective clothing for general care, double gloving, or placing protective covering for services that only involved casual touch. Excessive precautions enacted at the discretion of individual health care practitioners may have resulted from limited enforcement of universal health care precautions. However, these precautionary practices, when excessively or discriminately practiced may stigmatize people with HIV. These selective or discretionary uses of precautionary measures could also expose HIV-positive individuals to disclosure as well as to stigma.

#### Moral management

The literature discussed moral management within health care environments as a form of “judgementalism” [35] linked to the societal moralization of HIV infection. In these examples, health care practitioners were positioned as social judges perceived to be blaming patients for their infection. People with HIV discussed being moralized within health care environments in a number of ways: being ignored or infantilized, drawing disparaging comments, receiving neglectful care or being denied care due to a perceived denigrated lifestyle. Some studies also noted the power dynamic of health care centers, where the social positioning and professional status of health care practitioners permitted them to dictate “what is best for the patient” [69]. As remarked in Rutledge et al. (2009), this social positioning could contribute to providers using their societal power to engage in condemning practices while providing care at the same time [99]:

*“About two years ago I went to get a test for genital warts. . . . [T]here was . . . a male nurse. . . . He told me to pull down my pants and press my hands against the wall. He said, ‘Open your ass; I am going to test you’. So I did as he said, he shined the light and . . . the nurse decides to say, ‘Why you doing it with a man?! That is so wrong! . . . That’s why you get this thing now because you are not supposed to be buggering your ass!’ They put the medication on and said to come back in a week. . . . I am frightened for two years to go back and get the results of the test.” (Trinidadian MSM as quoted in Rutledge (2009), p. 24)*

Judgementalism of people with HIV often converged with other forms of societal moralization. Rajabiun et al. (2007) noted that some people with HIV attributed prejudicial treatment from health care practitioners to their historic or current drug use [94]:

*“When I went to the hospital, I told them I was HIV . . . he (the doctor) said, ‘So you are HIV and you use Crystal (meth).’ ‘Yes.’ And he looked down on me. And so he caused me a complex . . . he didn’t want to touch me.” (Unidentified participant quoted in Rajabiun (2007), p. S-24)*

Other studies noted inequitable care towards people who use drugs such as reluctance of providers to treat or to prescribe medications which people with HIV attributed to their drug use [71, 109].

The moral stratum to which certain people with HIV belong could also influence the quality of care they received and the level of stigma they endured. As discussed in Surlis (2001), perceived modes of HIV acquisition – drug use, sexual activity, transmission via blood transfusion, and vertical transmission – were morally stratified based on the level of fault attributed to the HIV-positive individual [106]. Women were particularly susceptible to moralization of their HIV-status: as sexually immoral if not partnered, as a vector of transmission if partnered, or as bad parents if expectant mothers or biological mothers of children with HIV [35, 82, 85, 87]. Moral judgements of people with HIV was indicative of symbolic stigma or the synergistic relationship between HIV-related stigma and the stigma attributed to the populations linked to HIV transmission [9]. Additionally, the moralization of people with HIV often intersected with dominant value systems: social role expectations, systems of privilege and oppression, and hierarchical constructions of social difference within broader society.

#### Intersectional stigma

HIV-related stigma was also expressed in relation to other forms of social marginalization. Study authors used terms such as “double stigma”, “multiple stigmas”, and “intensified stigma” to describe the interlinking of HIV-related stigma with other forms of marginalization due to race, ethnicity, socioeconomic status, class or caste, gender identity, sexual orientation or age. Thus, HIV-related stigma did not work in isolation; it was mutually constituted within other forms of marginalization to create interlocking matrices of oppression [115, 116].

As identified in Cain et al. (2001), the discrimination experienced by HIV-positive participants within health care environments seemed interlinked with other social prejudices [66]:

*“The hospital treats you bad. There are attitudes and gestures toward me being black, HIV positive, gay and a recovering drug user.” (African-American Male quoted in Cain (2001), p. 301)*

In some instances, these experiences of stigma within health care environments followed a “continuum of harm” [99] where participants ranked the manifestations of discriminatory behavior differently based on prior experiences of discrimination or the hiearchical positioning of one’s social group within broader society.

This use of terminology to illustrate the intersectional relationship between HIV-related stigma and other forms of marginalization echoes Parker & Aggleton’s assertion that conceptualization of stigma and HIV must consider broader notions of power, privilege and oppression [4]. HIV-related stigma played a key role in reproducing social difference: it designated people with HIV as the ‘social other’, and denoted the social, political and systemic factors that allowed power relations to occur between those who were stigmatized and those who enacted the stigma [88].

Experiences of enacted stigma within health care environments cross-cut the literature: it was identified in both high-income and low-income countries, and described in the earlier and the more recent studies. Synthesis findings indicate that discrimination within health care environments were a reflection of existing societal perceptions of HIV transmission, of people with HIV, and of activities and populations associated with HIV. Within the qualitative literature health care environments became another social space where perceptions of HIV and of people with HIV were demonstrated. Moreover, the health care environment existed as an institutional system where social values and mores, systems of power, and constructions of difference were instituted and operationalized. Consequently, stigmatizing attitudes against people living with HIV were demonstrated by those individuals and systems to whom people with HIV were expected to entrust their health.

### HIV, stigma and caring for one’s health as a person with HIV

Enacted, anticipated and felt stigma also played a role in caring for one’s health. The literature identified several stigma management strategies in which HIV-positive participants engaged to mitigate the negative effects of stigma. These included avoiding health care services where one’s status could be discerned and managing the “dilemma of visibility” [65] – signs and symptoms of HIV, opportunistic infections, or co-occurring illnesses – through health care practices. While some included studies identified stigma management strategies that could facilitate health and well-being, much of the qualitative evidence identified the detrimental effects of managing stigma through health care practices.

#### Managing stigma through health care utilization

The literature linked both felt and enacted stigma with health care utilization; fears of disclosure and anticipated stigma deterred some HIV-positive individuals from seeking treatment and utilizing care. Feelings of shame, blame, fear, and denial were also described as instrumental in delaying health care utilization.

Utilization practices were also devised as a means of avoiding stigma such as: using informal care, deffering disclosure of one’s status to health care practitioners, choosing larger medical centers, commuting to care outside of their community, and avoiding HIV-related health organizations, (i.e., HIV/AIDS service organization (ASOs), HIV/AIDS specialty care). Another form of stigma management included refusing treatment altogether [87]:

*“Today we had a very sick lady, and we wanted to give her IV fluids. . . . But she refused to stay here, simply because when she’s being visited, people will know she has HIV.” (Nurse quoted in Mill (2003), p. 12)*

Additionally, previous experiences of stigma within health care environments deterred utilization of care; these experiences of discrimination sometimes led individuals to avoid the clinic or hospital in question or to stay away from health care environments altogether, regardless of where it was delivered.

#### Managing stigma through adherence

Stigma also had a profound impact in achieving optimal adherence to antiretroviral treatments. As medication adherence could signify HIV-status, it subsequently became a form of inadvertent disclosure that could result in enacted stigma. Thus, medication use was discussed as being administered accordingly to manage potential disclosure. People with HIV engaged in various behaviors and actions to maintain status secrecy such as hiding medications in unmarked or alternative containers or disguising medications as breath mints or vitamins. As noted in Ingram et al. (1999), some participants would commonly use another illness believed to be less stigmatizing to explain their medication use [82]:

*“I just tell them that I got lymphoma. I guess we all sort of use the cancer theory.” (Unidentified participant quoted in Ingram (1999), p. 98)*

For some people with HIV, adherence and stigma avoidance became “competing priorities”, where they compromised adherence in order to avoid disclosure and anticipated stigma [110].

Adherence and stigma was a predominant theme in studies exploring the stigma experiences of children and youth with HIV. These studies identified the distinct adherence challenges for young people and the unique ways they and their caregivers had to navigate stigma while preserving young people’s sense of self. Anticipated stigma associated with the child’s adherence was a predominant concern for HIV-positive parents, especially for biological mothers of children with HIV. For mothers with HIV, adherence could elicit feelings of guilt, particularly for women who may blame themselves for their child’s HIV serostatus [111]:

*“Because he has the virus, since I gave it to him. If it wasn’t for the virus, he wouldn’t have to take it. And it reminds me every time I make him take the medicine. And I think that reminds him that he has the virus, so it bothers me”. (Biological mother of a 13-year-old boy quoted in Wrubel (2005), p. 2427)*

The stigma management strategies that caregivers utilized to shield their children from stigma were considered a form of “protective silence” [74], where caregivers managed adherence accordingly to avoid disclosure for fear of their child internalizing stigma, or to ensure their own status remained unknown. Yet, for young people unaware of their status, protective silence and adherence became particularly problematic. In cases where caregivers had not disclosed their child’s HIV serostatus to him or her, they had to be hypervigilant in maintaining optimal adherence without disclosing the purpose of the medication, sometimes resulting in parental force, child rebellion, or distrust [63]. Tippett Barr et al. (2007) explained that in cases where the caregiver had not disclosed the child’s status to other care supports, they became solely responsible for their child’s adherence [108]. This responsibility became challenging if they were unable to consistently care for their child’s health.

#### Impact of stigma management on health

Managing stigma through health care utilization or adherence could also have a deleterious impact on health including anxiety, substance misuse, depression and thoughts of suicide. For instance, depression related to HIV seropositivity was considered a significant barrier to maintaining optimal health, and potentially detrimental to accessing services and adhering to antiretroviral regimens. Felt stigma, as demonstrated through fear, self-isolation, or self-blame, could also contribute to depression.

Napravnik et al. (2000) noted that fear, sadness and guilt were particularly salient to the mental health of mothers with HIV [91]:

*“I was not ready to have a baby . . . [After] finding out about being pregnant [I] became bitter with myself. I cried a lot. I cried all the time. Because I felt it was just so unfair . . . Because here is a child, an innocent child, and you not know that, you know, she could be or he could be HIV-positive. You know? And it’s nothing that they did. They didn’t ask to come here. So, that was the main thing . . . I cried . . . I was very depressed during my pregnancy.“(“Janice” quoted in Napravnik (2000), p. 416)*

Fears of passing the infection to their children was identified as a potential barrier to accessing prenatal HIV care. Though expectant mothers may have wanted to ensure their children were born HIV negative, felt stigma intersecting with experience of sadness or distress as they navigate the health care system that challenged health care utilization and adherence to treatment.

Experiences of enacted or felt stigma also contributed to suicidal ideation [69, 100]. Some people with HIV offered suicide as a stigma management strategy, to avoid the “disfiguring features” associated with later stages of infection or as a way of keeping the diagnosis secret [107]. Sabin et al. (2008) indicated that in some cases, suicide became a means of “saving face” or to avoid shaming or burdening loved ones, particularly in family oriented cultures [100].

In some instances, substance use became a coping mechanism for HIV-related stigma. As noted in the mental health study conducted by the Committee for Accessible AIDS Treatment (2008), for some people with HIV, alcohol and drug use helped them cope with their status, along with other mental health concerns deriving from their HIV-diagnosis and subsequent stigma [70]:

*“I grew up with all these ideas about masculinity – men don't get depressed, men have to be aggressive, men don't have nervous breakdowns, men don't suffer from anxiety. To be a man is to be invincible. I started using drugs and alcohol … when I don't want to talk to people, when I am isolated, when I feel afraid of expressing my fears. Having to keep my feelings inside myself leads me to this self-destruction.” (Spanish-speaking man quoted in CAAT (2008), p. 19)*

While in some instance, substance use became a coping mechanism, in others it heightened experiences of felt stigma. Maher et al. (2007) noted that some people with HIV internalized the social moralization attributed to drug use. In their study, the label of *social evil*, a term ascribed to high-risk behaviors in Vietnam, was articulated in the narratives of study participants, where they referred to themselves as “social evils” [86]. Thus, their felt stigma and sense of self were an embodiment of the social devaluation of drug use and of HIV/AIDS.

For some people with HIV, stigma management through health care practices could provide some health benefits. Denial was a strategy that allowed some people to avoid the anxiety and stress stemming from enacted stigma. Denial through adherence − concealing use of antiretroviral medications or avoiding administration in public settings − could prevent disclosure of status. Avoiding health care settings could also mitigate status disclosure and subsequent stigma, a particularly salient concern for people who have had previous experiences of discrimination within health care environments. Geurtsen et al. (2005) indicated that for some people with HIV, avoiding stigma could be a means of preserving one’s sense of self, and a way to preserve dignity and a semblance of unaltered quality of life [78].

While there may exist some potential benefit of mitigating stigma through health care utilization and adherence practices, care avoidance practices as a stigma management strategy were more likely to have a negative impact on health. Not only could care avoidance exacerbate health concerns, but could also contribute to coping behaviors that put one’s health at further risk, or to self-preservation behaviors that denied instrumental and emotional support. While avoiding stigma could be considered by some people with HIV as a means of preserving quality of life, or saving face, it often prevented addressing stigma; additionally, it could facilitate its internalization.

### Strategies to address HIV-related stigma

A number of studies documented ways in which people with HIV addressed stigma and discrimination, including: social support, emotional support, practical support, and stigma-reducing interventions. These findings illustrate the many approaches people with HIV and others supporting their care can use to address stigma and its corresponding health impacts.

Social supports were key to addressing HIV-related stigma. People with HIV required support as they learned to live with the infection, adhere to medication regimens, access health care and community services, and resist the stigmatizing experiences they faced on a daily basis. Many people with HIV, particularly at the time of their diagnosis desired the emotional and practical support of the people close to them. They also made an effort to reach out and develop connections with others who were HIV-positive.

Lindau et al. (2006) indicated the importance of peer support for people with HIV, or gaining support from others who knew of the stigma they faced:

*“…sitting in the waiting room looking at all these people and all these thoughts were going through my head because I didn’t know it was a place for just HIV. I’m looking and I’m wondering and this lady came up to me, and she has been my friend ever since, and she said, ‘It’s not as bad as you think it is.’ I’m looking at her like you don’t know what my problem is. She looked at me and said ‘I have the same thing you’ve got and do I look like I’m dying?’ She was all nicely groomed and nicely dressed and she was going around smiling at everyone. And I said, ‘You don’t know what I got.’ And she said, ‘You got the virus, everybody here got the virus, that’s why we’re here.’ It is really important to get that personal interaction with other people in the same boat with you.” (Unidentified woman quoted in Lindau, (2006), p. 66)*

Some people with HIV found supportive environments with other people with HIV as fundamental to avoiding withdrawal and isolation. Alternatively, other people with HIV actively sought out connections with others outside of the HIV community as they could provide respite from the constant reminders of being HIV positive.

Families were also instrumental in the provision of emotional support. As noted by Sayles et al. (2007), some people with HIV reported their relationships with family strengthened following disclosure, which allowed them to view HIV as not just a challenge to be faced, but also as a motivator for positive change [102].

The literature also emphasized the importance of practical support for people living with HIV who may have experienced social isolation due to stigma. Community programs provided important support systems for people with HIV, at times filling in the gaps where partners, friends, and family could not or would not offer the necessary assistance. Compassionate health care providers also served as an essential component of the support systems of people with HIV. Rajabiun et al. (2007) indicated that relationships with service providers could even become the primary source of support [94]:

*“Patient advocates, social workers, [everyone in this program] it’s good to have. Because I go through a lot of situations, like I said, I got fired . . . And it’s good to have someone you can call to help you. They’re patient. They will actually sit down and talk to you. They will not blow you off. I think if it weren’t for [the outreach program] I’ll probably still be out here in the streets, doing the same thing, if it weren’t for this organization.” (Unidentified participant quoted in Rajabiun (2007), p. S-27)*

Non-judgmental attitudes from health care workers was identified as fundamental to creating a safe space for people with HIV. Supportive attitudes from health care workers was also discussed as facilitating health: encouraging care seeking behaviors, motivating adherence, encouraging communication, and decreasing social isolation and exclusion.

Also identified were a variety of approaches to actively resist and reduce stigma and discrimination. The most common was involving people with HIV in stigma reduction strategies. For instance, people with HIV served as self-advocates by raising awareness, educating others in their communities, or through formal volunteerism within ASOs. Self-advocacy also allowed people with HIV to move away from denial about the diagnosis and towards taking care of their health. As offered by Sayles (2007), in communities where the threat of disclosure was great, merely sharing one’s status openly was viewed by people with HIV as an act of resistance [102]:

*“What I did was I empowered myself by disclosing. I went on TV, radio talk shows, and magazines.... I just went public with my whole life and I felt like a sense of freedom and I gained my power. I am comfortable with the facts and I am very realistic that I have this virus in my body.... Right now there is no way I can get rid of it, so therefore I must learn to live with it. I am going to live in this world whether you like me or not. This is my world.” (Unidentified female participant quoted in Sayles (2007), p. 821)*

Furthermore, the qualitative literature demonstrated that people with HIV could play a significant role in collective advocacy. The Committee for Accessible AIDS Treatment (2008) offered the active involvement and engagement of “PHAs” or people with HIV as instrumental to HIV movements [70]:

*“I think we need PHA leaders… it's those early pioneers who admitted that they were survivors of the mental health service industry and came forward and really challenged us and other social service organizations to develop. I really feel strongly that there needs to be leaders in the IRN-PHA community who come forth. It's an unfortunate burden that they have to bear, it's not very equitable, but I think that's important.” (Ethno-specific ASO male quoted in CAAT (2008), p. 33)*

Other community members also acted as advocates in stigma reduction strategies. Family, friends, health care and social service practitioners were offered as key players in proclaiming support for people with HIV and in raising awareness among service providers and the general community.

Synthesis findings illustrate that in certain conditions, people with HIV can transform their experiences of stigma in ways that create opportunities for empowerment and change [117]. Notably, many of these strategies require the support of others impacted by HIV: friends, families, partners and the greater communities.

## Discussion

This synthesis of the qualitative evidence exploring stigma, HIV and health demonstrates that HIV-related stigma within the health context is reflective of the society at large, and that it is socially embedded, shaped, and influenced by social and cultural mores and values, a finding substantiated in other reviews ﻿[11, 39, 135]. Exclusively reviewing the qualitative literature offers a nuanced understanding of the manifestation of stigma within health contexts. Particularly, it illustrates the emotional, attitudinal, and structural dimensions of stigma as experienced by people with HIV.

While HIV-related stigma within health care settings has been attributed to misperceptions surrounding transmission, findings from this synthesis also indicate that emotions serve as a driver of HIV-related stigma. The affective dimensions of stigma shaped practitioner care practices even when knowledge of HIV transmission risks were available. While other reviews have noted that practitioners’ fear of casual contact may stem from limited information on HIV transmission risks [11], findings from this review also illustrate how fears can arise in practitioners who are knowledgeable of the risks. These findings emphasize the psychosocial nature of stigma and discrimination. Prejudice is a prejudgement that has both cognitive and affective dimensions [120]. To prevent a prejudgement from manifesting as discriminatory treatment, knowledge provision must attend to both its cognitive and affective aspects as emotions can overpower rational decision making. While education has served to mitigate some of the misperceptions surrounding HIV, there still remains the affective dimensions that contribute to discriminatory behaviours such as fear of transmission through the provision of care. These findings suggest that stigma reduction interventions for health care practitioners must do more than relay knowledge, they also must acknowledge and address the emotional aspects as well.

Along with the affective dimensions of stigma, there also exists the attitudinal or behavioural dimensions. These dimensions of stigma are best demonstrated when looking at the literature exploring the moral aspects of stigma. Particularly, synthesis findings illustrate the interconnections between moral attributions and the devaluations of social groups disproportionately impacted by HIV. Moral attributions become modes of enforcing dominant social values, including dominant constructions of sexuality, substance use, sex work, and mothering [88]. Additionally, HIV-related stigma ascribed to activities deemed socially deviant consequently attach to those who engage in such activities [121]. Thus, the moralization of HIV and its transmission behaviours further marginalized populations that are devalued in broader society while rationalizing discriminatory behaviours through attribution of blame [122].

As well, this synthesis illustrates the complexities of intersectional stigma particularly when juxtaposed with moralization of transmission activities such as drug use, sex work, or sexual activities outside of heterosexual, monogamous relationships. While Herek’s conceptualization of symbolic stigma [8, 9] offers a framework for understanding moralized activities begetting moralized identities, this review emphasizes the synergistic connection between HIV-related stigma and pre-existing prejudices including sexism, cis-genderism/transphobia, heterosexism/homophobia, racism, and Eurocentrism. Intersectional stigma within the context of this review signifies the dynamic social processes of marginalization that are demonstrative of cultural values, mores and social power [4]. As the existing literature on sexuality, race, and gender discriminations have emphasized, social marginalization of populations experiencing discrimination in general society correlates with adverse health outcomes [123–129], many of which parallel those correlating with HIV-related stigma. While there is a preponderance of research examining singular forms of oppressions and its impacts on health, only recently has research started to examine intersectional stigma in the context of HIV-seropositivity [130–132]. More research examining stigma, HIV and health from an intersectional perspective is warranted given the emerging findings from this review.

While enacted stigma can have a profound impact on health care access, our synthesis identifies felt stigma as impactful on the health and well-being of people with HIV. Consistent with the quantitative literature, avoiding HIV-related stigma was often presented as being detrimental to health [52, 53]. However, the qualitative literature also noted that in some circumstances, avoiding stigma could be a means of mitigating its effects. These varied outcomes highlight the potential competing priorities people with HIV may face when managing their health. Managing stigma through avoiding HIV-related health care practices can be perceived as having a potential immediate benefit to one’s mental or emotional well-being such as reduced stress stemming from anticipated stigma; yet, it can consequently contribute to deleterious health outcomes in the long term [110]. Additionally, some people with HIV may have to balance competing priorities between individual and collective need, where they may engage in practices to avoid stigma in order to save face within their community, protect their HIV-positive children from disclosure, or to protect their loved ones from worry or secondary stigma [86, 100]. These synthesis findings illustrate the importance of examining qualitative evidence collectively to understand the nuanced nature of stigma management and the interpersonal, intrapersonal, and social contexts which shape health care decision making of people with HIV.

Conversely, this synthesis demonstrates that under certain conditions, people with HIV may combat or transform their stigma experiences in ways that create opportunities for empowerment, resistance and social change [117]. Notably, many of the strategies identified as addressing stigma require the support of others affected by HIV: friends, families, partners, communities, and advocates within health care environments. These findings indicate that stigma management is not a sole effort, but a collective and communal one as well. Other literature has also indicated the importance of involving people with HIV in stigma reduction responses and incorporating community interventions in stigma reduction campaigns [133].

Lastly, this synthesis of the qualitative evidence illustrates that HIV-related stigma within health contexts is a global phenomenon for people with HIV. While the dimensions of stigma were similar across studies, findings from qualitative studies indicate that the manifestations of stigma within health domains are temporally and culturally shaped. For instance, in the earlier studies, the terminality of HIV and the fear of contagion seemed more prominent in discriminatory health care practices than in the more recent studies, a finding that has been substantiated in other literature [134]. Interpretations of stigma also differed across geographic jurisdictions. For example, disclosure of status to family was a more typical confidentiality violation in non-Western cultures, particularly African and Asian cultures where family unity is more culturally bound [73, 88, 100]. Yet, studies in Western cultures such as Europe, Canada and the United States, also reported examples of confidentiality violations, particularly processes that were institutionalized or integrated into practice such as distinguishing labels on personal records, tests or medications, or practitioners discussing client cases in non-private spaces [60, 74].

Despite differences in the interpretation of stigma experiences, the identified cases were fairly similar across studies with examples in health care access, utilization, adherence, and mental health, a finding also identified in another literature review [135]. More notably, extreme examples of stigma within health care such as blatant disregard for confidentiality, degrading comments, humiliating treatment, and denial of care were identified across temporal and geographic contexts, including more recent studies conducted in countries such as Canada and the United States, where stigma is a focal point of HIV education and modes of transmission are commonly promoted to the general public. While HIV-related stigma is a global phenomenon, future research on HIV-related stigma and stigma reduction strategies should consider local manifestation of stigma within the health context as well as how stigma can be differentially experienced across cultures.

Findings from this qualitative synthesis indicate that combating HIV-related stigma within health care environments and addressing the health-related effects will require more than targeted interventions directed towards health care practitioners or individuals with HIV. While stigma reducing interventions for people with HIV can mitigate some individual health effects of felt stigma, they do not address its social embeddedness which fosters enacted stigma. While HIV education may address overt forms of HIV-related discrimination, it cannot illustrate the subtle ways that activities within health care settings can be stigmatizing nor will it address the structural dimensions of stigma within society at large. Some of the challenges of combating HIV-related stigma within health care settings stem from structural factors such as discrimination of people with HIV within other social spheres, limited legislation prohibiting discrimination of people with HIV, and discriminatory practices embedded within jurisdictional legislation [136]. Recent evidence emphasizes that stigma reduction interventions within health care settings should also address discrimination within institutional culture as well as factors that foster HIV-related stigma at the individual, environmental and societal levels [137]. Furthermore, HIV-related stigma intersects with many other forms of marginalization − sexual identity, gender identity, race, ethnicity, socioeconomic status, class or caste, for example. Undertaking intersectional stigma requires a multidimensional approach in order to address its mutual constitutiveness. Review findings demonstrate that tackling HIV-related stigma will require acknowledgement and address of other forms of discrimination that disproportionately intersect with HIV.

Therefore, findings of this synthesis indicate that to reduce HIV-related stigma − the third phase of the HIV epidemic identified twenty-five years ago that still exists to this day − local and global interventions, and future stigma research will need to consider the social structures and societal practices, within and outside of health care environments, that perpetuate and reinforce stigma and discrimination towards people with HIV.

### Strengths and limitations of this synthesis

This qualitative synthesis performed a comprehensive search of the peer-reviewed literature, used a consensus approach to select and extract data from relevant papers, and presented detailed information of included studies and thematically summarized the findings. As with any other systematic review, it also had a few limitations. In the analysis we were unable to include studies published in languages other than English, French or Spanish. The search strategy did not include an electronic database search for grey literature, although we did conduct manual searches that could capture non-peer reviewed or unpublished literature. Even though the included findings were geographically diverse, study definitions derived from Westernized knowledge of stigma and health, and may not have aptly captured non-Western or Indigenous conceptualizations of these terms.

## Conclusions

The synthesis of the qualitative evidence identified 55 qualitative studies that illustrate HIV-related stigma in the context of health. HIV-related stigma is a psychological and social process in which HIV-positive individuals struggle to cope with the misperceptions, social separation, denigration, and discriminatory actions associated with their status. Findings from this synthesis indicate that HIV-related stigma is a global social phenomenon for people with HIV that manifests within multiple social spheres, including health care environments. The qualitative literature identifies a number of strategies used within health care settings − some rooted in institutional practices, others shaped by personal perceptions held by practitioners − that could subsequently be stigmatizing or discriminatory for people with HIV. The literature also identifies strategies people with HIV may use to manage stigma as they utilize health care or adhere to treatment regimes. While some stigma management strategies may mitigate the mental health impacts of stigma, others may put one’s health at risk. These health care strategies related to HIV and stigma suggest the dynamic interconnections of enacted and felt stigma along with marginalization, moralization, disclosure, and the visibility of HIV/AIDS as a health condition.

Findings from qualitative evidence also suggest modes of addressing stigma that can disrupt this cyclical process. The literature identifies a number of ways to address stigma and its health impacts including social support, education, self-efficacy and resilience strategies, and social and individual advocacy. Since stigma and discrimination incorporates cognitive, affective, attitudinal and behavioural dimensions and is experienced and enacted at individual, community, and societal levels, multi-dimensional, multi-level societal responses to HIV-related stigma are required to address all of these aspects. While people impacted by HIV should shape stigma reduction strategies, it will take a collective, societal response to combat HIV-related stigma and its health effects.

## Endnotes

^1^Of the 11 foreign language papers, four each were published in Chinese and Portuguese, and one each were published in Czech, German, and Serbian.

## Additional files

Additional file 1:
**Search strategies used for stigma reviews.** This file details the electronic and manual search strategies conducted for this review. (PDF 23 kb) Additional file 2:
**Articles excluded from qualitative data extraction.** This file provides a reference list of papers excluded from the qualitative synthesis at data extraction (i.e. studies that reported only quantitative findings, and studies published in other languages than English, French or Spanish). (PDF 24 kb) Additional file 3:
**Summary of study characteristics.** This file summarizes key characteristics of included studies (i.e., study aims/objectives, geographic jurisdiction, characteristics of study sample, and concept of stigma applied in the study). (PDF 37 kb) Additional file 4:
**Matrix summary of qualitative themes.** This file classifies the common themes related to HIV, stigma and health as identified in the included studies. (PDF 81 kb) Additional file 5:
**Descriptive summary of themes.** This files defines the common themes identified in the qualitative literature related to HIV, stigma and health. (PDF 17 kb)

## References

[1] Mann JM (1988). Statement at an Informal Briefing on AIDS to the 42nd Session of the United Nations General Assembly. Journal of the Royal Statistical Society Series A (Statistics in Society)..

[2] Goffman E (1963). Stigma: Notes on the Management of Spoiled Identity.

[3] Alonzo AA, Reynolds NR (1995). Stigma, HIV and AIDS: An exploration and elaboration of a stigma trajectory. Soc Sci Med.

[4] Parker R, Aggleton P (2003). HIV and AIDS-related stigma and discrimination: A conceptual framework and implications for action. Soc Sci Med.

[5] Taylor B (2001). HIV, stigma and health: Integration of theoretical concepts and the lived experiences of individuals. J Adv Nurs.

[6] Earnshaw V, Chaudoir S (2009). From Conceptualizing to Measuring HIV Stigma: A Review of HIV Stigma Mechanism Measures. AIDS Behav.

[7] Holzemer WL, Uys L, Makoae L, Stewart A, Phetlhu R, Dlamini PS (2007). A conceptual model of HIV/AIDS stigma from five African countries. J Adv Nurs.

[8] Herek GM, Capitanio JP (1998). Symbolic prejudice or fear of infection? A functional analysis of AIDS-related stigma among heterosexual adults. Basic and Applied Social Psychology.

[9] Herek GM (1999). AIDS and stigma. Am Behav Sci.

[10] Collins E, Cain R, Bereket T, Chen YY, Cleverly S, George C (2007). Living & Serving II: 10 Years Later - The Involvement of People Living with HIV/AIDS in the Community AIDS Movement in Ontario.

[11] Nyblade L, Stangl A, Weiss E, Ashburn K (2009). Combating HIV stigma in health care settings: What works?. J Int AIDS Soc.

[12] Sears B, Cooper C, Younai FS, Donohoe T (2012). HIV discrimination in dental care: Results of a testing study in Los Angeles county. Loyola Los Angel Law Rev.

[13] Elford J, Ibrahim F, Bukutu C, Anderson J (2008). HIV-related discrimination reported by people living with HIV in London. UK. AIDS Behav..

[14] Schuster MA, Collins R, Cunningham WE, Morton SC, Zierler S, Wong M (2005). Perceived discrimination in clinical care in a nationally representative sample of HIV‐infected adults receiving health care. J Gen Intern Med.

[15] Fife BL, Wright ER. The dimensionality of stigma: A comparison of its impact on the self of persons with HIV/AIDS and cancer. J Health Soc Behav. 2000;50–67. 10750322

[16] Sandelowski M, Lambe C, Barroso J (2004). Stigma in HIV-Positive Women. J Nurs Scholarsh.

[17] Gonzalez A, Solomon SE, Zvolensky MJ, Miller CT (2009). The interaction of mindful-based attention and awareness and disengagement coping with HIV/AIDS-related stigma in regard to concurrent anxiety and depressive symptoms among adults with HIV/AIDS. J Health Psychol.

[18] Ivanova E, Hart T, Wagner A, Aljassem K, Loutfy M. Correlates of Anxiety in Women Living with HIV of Reproductive Age. AIDS Behav. 2012:1–11. doi:10.1007/s10461-011-0133-6. 10.1007/s10461-011-0133-6 22246517

[19] Wagner AC, Hart TA, Mohammed S, Ivanova E, Wong J, Loutfy MR (2010). Correlates of HIV stigma in HIV-positive women. Arch Womens Ment Health.

[20] Rueda S, Gibson K, Rourke S, Bekele T, Gardner S, Cairney J (2012). Mastery Moderates the Negative Effect of Stigma on Depressive Symptoms in People Living with HIV. AIDS Behav.

[21] Lee SL, Kochman A, Sikkema KJ (2002). Internalized stigma among people living with HIV-AIDS. AIDS Behav.

[22] Peltzer K, Ramlagan S (2011). Perceived stigma among patients receiving antiretroviral therapy: a prospective study in KwaZulu-Natal. South Africa. AIDS Care..

[23] Capron DW, Gonzalez A, Parent J, Zvolensky MJ, Schmidt NB (2012). Suicidality and Anxiety Sensitivity in Adults with HIV. AIDS Patient Care STDS.

[24] Carrico AW (2010). Elevated suicide rate among HIV-positive persons despite benefits of antiretroviral therapy: implications for a stress and coping model of suicide. Am J Psychiatry.

[25] Vance D (2006). Self-rated emotional health in adults with and without HIV. Psychol Rep.

[26] Varni SE, Miller CT, McCuin T, Solomon S (2012). Disengagement and engagement coping with HIV/AIDS stigma and psychological well-being of people with HIV/AIDS. J Soc Clin Psychol.

[27] Greeff M, Uys LR, Wantland D, Makoae L, Chirwa M, Dlamini P (2010). Perceived HIV stigma and life satisfaction among persons living with HIV infection in five African countries: A longitudinal study. Int J Nurs Stud.

[28] Vyavaharkar M, Moneyham L, Murdaugh C, Tavakoli A (2012). Factors associated with quality of life among rural women with HIV disease. AIDS Behav.

[29] Holzemer WL, Human S, Arudo J, Rosa ME, Hamilton MJ, Corless I (2009). Exploring HIV stigma and quality of life for persons living with HIV infection. J Assoc Nurses AIDS Care.

[30] Dlamini PS, Wantland D, Makoae LN, Chirwa M, Kohi TW, Greeff M (2009). HIV stigma and missed medications in HIV-positive people in five African countries. AIDS Patient Care STDS.

[31] Kinsler JJ, Wong MD, Sayles JN, Davis C, Cunningham WE (2007). The effect of perceived stigma from a health care provider on access to care among a low-income HIV-positive population. AIDS Patient Care STDS.

[32] Golin C, Isasi F, Bontempi JB, Eng E (2002). Secret pills: HIV-positive patients' experiences taking antiretroviral therapy in North Carolina. AIDS Educ Prev.

[33] Rao D, Feldman BJ, Fredericksen RJ, Crane PK, Simoni JM, Kitahata MM (2012). A structural equation model of HIV-related stigma, depressive symptoms, and medication adherence. AIDS Behavior.

[34] Mann JM, Tarantola D, editors. AIDS in the world II. Oxford, UK: Oxford University Press; 1996.

[35] Brickley DB (2007). AIDS stigma and women: Impact on prevention and treatment interventions in Vietnam in the era of antiretroviral therapy.

[36] Valdiserri RO (2002). HIV/AIDS stigma: an impediment to public health. Am J Public Health.

[37] Kingori C, Reece M, Obeng S, Murray M, Shacham E, Dodge B (2012). Impact of internalized stigma on HIV prevention behaviors among HIV-infected individuals seeking HIV care in Kenya. AIDS Patient Care STDS.

[38] Vanable PA, Carey MP, Blair DC, Littlewood RA (2006). Impact of HIV-related stigma on health behaviors and psychological adjustment among HIV-positive men and women. AIDS Behav.

[39] Brown L, Macintyre K, Trujillo L (2003). Interventions to reduce HIV/AIDS stigma: what have we learned?. AIDS Educ Prev.

[40] Crawford AM (1996). Stigma associated with AIDS: A meta-analysis. Journal of Applied Social Psychology.

[41] Mak WWS, Poon CYM, Pun LYK, Cheung SF (2007). Meta-analysis of stigma and mental health. Soc Sci Med.

[42] Vidanapathirana J, Randeniya M, Operario D (2007). Interventions for reduction of stigma in people with HIV/AIDS (Protocol). Cochrane Database Syst Rev..

[43] Torrence H. Qualitative research, science, and government: Evidence, criteria, policy, and politics. In: Denzin NK, Lincoln YS, editors. The SAGE handbook of qualitative research. 4th ed. Thousand Oaks, CA: Sage Publication; 2011.p. 569-80.

[44] Hannes K, Macaitis K. A move to more systematic and transparent approaches in qualitative evidence synthesis. Qual Res. 2010.

[45] Noyes J, Popay J, Pearson A, Hannes K, Booth A, The Cochrane Qualitative Research Methods Group. Qualitative research and Cochrane reviews. In: Higgins J, Green S, editors. Cochrane Handbook for Systematic Reviews of Interventions. 5.1.0 ed: The Cochrane Collaboration; 2011.

[46] Sandelowski M, Barroso J (2007). Handbook for synthesizing qualitative research.

[47] Popay J. Incorporating qualitative information in systematic reviews. 14th Cochrane Colloquium; Dublin, Ireland2006.

[48] Campbell R, Pound P, Pope C, Britten N, Pill R, Morgan M (2003). Evaluating meta-ethnography: a synthesis of qualitative research on lay experiences of diabetes and diabetes care. Soc Sci Med.

[49] Barroso J, Powell-Cope GM (2000). Metasynthesis of qualitative research on living with HIV infection. Qual Health Res.

[50] Walsh D, Downe S (2005). Meta-synthesis method for qualitative research: A literature review. J Adv Nurs.

[51] Emlet CA (2006). An examination of the social networks and social isolation in older and younger adults living with HIV/AIDS. Health Soc Work.

[52] Logie C, Gadalla T (2009). Meta-analysis of health and demographic correlates of stigma towards people living with HIV. AIDS Care.

[53] Rueda S, Mitra S, Chen S, Gogolishvili D, Globerman J, Chambers LA et al. Examining the associations between HIV-related stigma and health outcomes in people living with HIV/AIDS: a meta-analysis. AIDS Behav. 2014;under review. 10.1136/bmjopen-2016-011453 PMC4947735 27412106

[54] Link BG, Phelan JC (2001). Conceptualizing stigma. Annu Rev Sociol.

[55] Sandelowski M, Barroso J (2003). Creating metasummaries of qualitative findings. Nurs Res.

[56] McInnes RJ, Chambers JA (2008). Supporting breastfeeding mothers: qualitative synthesis. J Adv Nurs.

[57] Braun V, Clarke V (2006). Using thematic analysis in psychology. Qualitative research in psychology.

[58] Patton MQ (1990). Qualitative evaluation and research methods.

[59] Agne RR, Thompson TL, Cusella LP (2000). Stigma in the line of face: Self-disclosure of patients' HIV status to health care providers. Journal of Applied Communication Research..

[60] Anderson M, Elam G, Gerver S, Solarin I, Fenton K, Easterbrook P (2008). HIV/AIDS-related stigma and discrimination: accounts of HIV-positive Caribbean people in the United Kingdom. Soc Sci Med.

[61] Balabanova Y, Coker R, Atun RA, Drobniewski F (2006). Stigma and HIV infection in Russia. AIDS Care.

[62] Barnes DB, Alforque A, Carter K (2000). "Like I Just Got a Death Sentence": Conditions Affecting Women's Reactions to Being Told Their HIV Antibody Test Results and the Impact on Access to Care. Res Sociol Health Care..

[63] Bikaako-Kajura W, Luyirika E, Purcell DW, Downing J, Kaharuza F, Mermin J (2006). Disclosure of HIV status and adherence to daily drug regimens among HIV-infected children in Uganda. AIDS Behav.

[64] Brion JM (2007). Perspectives regarding adherence to prescribed treatment: a focus group study of HIV positive men: Ohio State University.

[65] Buseh AG, Stevens PE, McManus P, Addison J, Morgan S, Millon-Underwood S (2006). Challenges and opportunities for HIV prevention and care: insights from focus groups of HIV-infected African American men. J Assoc Nurses AIDS Care.

[66] Cain RE (2001). Quality of life issues among a small sample of persons living with HIV disease in a rural area. Int Electron J Health Educ..

[67] Cao X, Sullivan SG, Xu J, Wu Z (2006). Understanding HIV-Related Stigma And Discrimination in a Blameless Population. AIDS Educ Prev.

[68] Carr RL, Gramling LK (2004). Stigma: A health barrier for women with HIV/AIDS. J Assoc Nurses AIDS Care.

[69] Castro R, Orozco E, Eroza E, Manca MC, Hernandez JJ, Aggleton P (1998). AIDS-related illness trajectories in Mexico: findings from a qualitative study in two marginalized communities. AIDS Care.

[70] Committee for Accessible AIDS Treatment. Transformation through collective action: Best practices in migration, HIV and mental health. Toronto, ON: CAAT2008.

[71] Dawson-Rose C, Shade SB, Lum PJ, Knight KR, Parsons JT, Purcell DW (2005). Health care experiences of HIV positive injection drug users. J Multicult Nurs Health.

[72] Edwards LV (2006). Perceived social support and HIV/AIDS medication adherence among African American women. Qual Health Res.

[73] Elamon J (2005). A situational analysis of HIV/AIDS-related discrimination in Kerala. India. AIDS Care..

[74] Emlet CA (2007). Experiences of stigma in older adults living with HIV/AIDS: a mixed-methods analysis. AIDS Patient Care STDS.

[75] Erwin J, Peters B (1999). Treatment Issues for HIV+ Africans in London. Soc Sci Med..

[76] Gardezi F, Calzavara L, Husbands W, Tharao W, Lawson E, Myers T (2008). Experiences of and responses to HIV among African and Caribbean communities in Toronto. Canada. AIDS Care..

[77] Gaudine A, Gien L, Thuan TT, Dung DV (2007). Perspectives of the stigma of HIV from one community in Vietnam: A qualitative descriptive study. Int J Nurs Stud.

[78] Geurtsen B (2005). Quality of life and living with HIV/AIDS in Cambodia. J Transcult Nurs.

[79] Greeff M, Phetlhu R (2007). The meaning and effect of HIV/AIDS stigma for people living with AIDS and nurses involved in their care in the North West Province. South Africa. Curationis..

[80] Green G, Platt S (1997). Fear and Loathing in Health Care Settings Reported by People with HIV. Sociol Health Illn.

[81] Herrera C, Campero L, Caballero M, Kendall T (2008). Relationship between physicians and HIV patients: influence on adherence and quality of life. [Spanish]. Rev Saude Publica.

[82] Ingram D, Hutchinson SA (1999). HIV-positive mothers and stigma. Health Care Women Int.

[83] Konkle-Parker DJ, Erlen JA, Dubbert PM (2008). Barriers and facilitators to medication adherence in a southern minority population with HIV disease. J Assoc Nurses AIDS Care.

[84] Kumarasamy N, Safren SA, Raminani SR, Pickard R, James R, Sri Krishnan AK (2005). Barriers and facilitators to antiretroviral medication adherence among patients with HIV in Chennai, India: a qualitative study. AIDS Patient Care STDS.

[85] Lindau ST, Jerome J, Miller K, Monk E, Garcia P, Cohen M (2006). Mothers on the margins: implications for eradicating perinatal HIV. Soc Sci Med.

[86] Maher L, Coupland H, Musson R (2007). Scaling up HIV treatment, care and support for injecting drug users in Vietnam. Int J Drug Policy.

[87] Mill JE (2003). Shrouded in secrecy: breaking the news of HIV infection to Ghanaian women. J Transcult Nurs.

[88] Mills EA (2006). From the Physical Self to the Social Body: Expressions and Effects of HIV-Related Stigma in South Africa. J Community Appl Soc Psychol..

[89] Murray LK, Semrau K, McCurley E, Thea DM, Scott N, Mwiya M (2009). Barriers to acceptance and adherence of antiretroviral therapy in urban Zambian women: A qualitative study. AIDS Care.

[90] Muyinda H, Seeley J, Pickering H, Barton T (1997). Social aspects of AIDS-related stigma in rural Uganda. Health Place.

[91] Napravnik S, Royce R, Walter E, Lim W (2000). HIV-1 infected women and prenatal care utilization: Barriers and facilitators. AIDS Patient Care STDS.

[92] Nguyen TA, Oosterhoff P, Ngoc YP, Wright P, Hardon A. Barriers to access prevention of mother-to-child transmission for HIV positive women in a well-resourced setting in Vietnam. AIDS Res Ther. 2008;5(7):doi:10.1186/742-6405-5-7. 10.1186/1742-6405-5-7 PMC2358919 18419808

[93] Pugatch D, Bennett L, Patterson D (2002). HIV medication adherence in adolescents: a qualitative study. J HIV/AIDS Prev Educ Adolesc Child.

[94] Rajabiun S, Mallinson RK, McCoy K, Coleman S, Drainoni M, Rebholz C (2007). "Getting me back on track": the role of outreach interventions in engaging and retaining people living with HIV/AIDS in medical care. AIDS Patient Care STDS..

[95] Rao D, Kekwaletswe TC, Hosek S, Martinez J, Rodriguez F (2007). Stigma and social barriers to medication adherence with urban youth living with HIV. AIDS Care.

[96] Rintamaki LS, Scott AM, Kosenko KA, Jensen RE (2007). Male patient perceptions of HIV stigma in health care contexts. AIDS Patient Care STDS.

[97] Roberson DW (2009). Factors influencing adherence with anti-retroviral therapy for HIV positive female inmates. J Assoc Nurses AIDS Care.

[98] Roberts KJ (2005). Barriers to Antiretroviral Medication Adherence in Young HIV-Infected Children. Youth Soc.

[99] Rutledge SE, Abell N, Padmore J, McCann TJ (2009). AIDS stigma in health services in the Eastern Caribbean. Sociol Health Illn.

[100] Sabin LL, DeSilva MB, Hamer DH, Keyi X, Yue Y, Wen F (2008). Barriers to adherence to antiretroviral medications among patients living with HIV in southern China: A qualitative study. AIDS Care.

[101] Sanjobo N, Frich JC, Fretheim A (2008). Barriers and facilitators to patients' adherence to antiretroviral treatment in Zambia: A qualitative study. SAHARA J.

[102] Sayles JN, Ryan GW, Silver JS, Sarkisian CA, Cunningham WE (2007). Experiences of social stigma and implications for healthcare among a diverse population of HIV positive adults. J Urban Health.

[103] Schilder AJ, Kennedy C, Goldstone IL, Ogden RD, Hogg RS, O'Shaughnessy MV (2001). "Being dealt with as a whole person." Care seeking and adherence: The benefits of culturally competent care. Soc Sci Med.

[104] Starks H, Simoni J, Zhao H, Huang B, Fredriksen-Goldsen K, Pearson C (2008). Conceptualizing antiretroviral adherence in Beijing. China. AIDS Care..

[105] Steward WT, Herek GM, Ramakrishna J, Bharat S, Chandy S, Wrubel J (2008). HIV-related stigma: adapting a theoretical framework for use in India. Soc Sci Med.

[106] Surlis S, Hyde A (2001). HIV-positive patients' experiences of stigma during hospitalization. J Assoc Nurses AIDS Care.

[107] Thi MDA, Brickley DB, Vinh DTN, Colby DJ, Sohn AH, Trung NQ et al. A qualitative study of stigma and discrimination against people living with HIV in Ho Chi Minh City, Vietnam. AIDS Behav. 2008;12(4): Supplement. 10.1007/s10461-008-9374-4 18360743

[108] Tippett Barr BA (2007). Pediatric antiretroviral adherence and child disclosure in Botswana.

[109] Wang Y, Zhang KN, Zhang KL (2008). HIV/AIDS related discrimination in health care service: A cross-sectional study in Gejiu City. Yunnan Province. Biomed Environ Sci..

[110] Ware N, Wyatt M, Tugenberg T (2006). Social relationships, stigma and adherence to antiretroviral therapy for HIV/AIDS. AIDS Care.

[111] Wrubel J, Moskowitz JT, Richards TA, Prakke H, Acree M, Folkman S (2005). Pediatric adherence: Perspectives of mothers of children with HIV. Soc Sci Med.

[112] Zukoski AP, Thorburn S (2009). Experiences of stigma and discrimination among adults living with HIV in a low HIV-prevalence context: A qualitative analysis. AIDS Patient Care STDS.

[113] UNAIDS. Protocol for the identification of discrimination against people living with HIV. http://data.unaids.org/Publications/IRC-pub01/jc295-protocol_en.pdf. Geneva, SW2000.

[114] Li L, Liang LJ, Lin C, Wu Z, Wen Y (2009). Individual attitudes and perceived social norms: Reports on HIV/AIDS-related stigma among service providers in China. Int J Psychol.

[115] Crenshaw K. Mapping the margins: Intersectionality, identity politics, and violence against women of color. In: Grewal I, Kaplan C, editors. An Introduction to Women's Studies: Gender in a Transnational World. 2nd Edition ed. Boston: McGraw Hill; 2002. p. 207–13.

[116] Collins PH (1998). Intersections of race, class, gender, and nation: Some implications for Black family studies. Journal of Comparative Family Studies.

[117] Howarth C (2006). Race as stigma: Positioning the stigmatized as agents, not objects. J Community Appl Soc Psychol.

[118] Rogers S, Tureski K, Cushnie A, Brown A, Bailey A, Palmer Q (2014). Layered stigma among health-care and social service providers toward key affected populations in Jamaica and The Bahamas. AIDS Care.

[119] Rutledge SE, Whyte J, Abell N, Brown KM, Cesnales NI (2011). Measuring stigma among health care and social service providers: The HIV/AIDS Provider Stigma Inventory. AIDS Patient Care STDS.

[120] Allport GW (1979). The nature of prejudice.

[121] Madru N (2003). Stigma and HIV: does the social response affect the natural course of the epidemic?. J Assoc Nurses AIDS Care.

[122] Herek GM, Widaman KF, Capitanio JP (2005). When sex equals AIDS: Symbolic stigma and heterosexual adults' inaccurate beliefs about sexual transmission of AIDS. Soc Probl.

[123] Williamson IR (2000). Internalized homophobia and health issues affecting lesbians and gay men. Health Educ Res.

[124] Newcomb ME, Mustanski B (2010). Internalized homophobia and internalizing mental health problems: A meta-analytic review. Clin Psychol Rev.

[125] Shavers VL, Shavers BS (2006). Racism and health inequity among Americans. J Natl Med Assoc.

[126] Brondolo E, Gallo L, Myers H (2009). Race, racism and health: disparities, mechanisms, and interventions. J Behav Med.

[127] Paradies Y (2006). A systematic review of empirical research on self-reported racism and health. Int J Epidemiol.

[128] Klonoff EA, Landrine H (1995). The schedule of sexist events. Psychology of Women Quarterly.

[129] Krieger N, Rowley DL, Herman AA, Avery B, Phillips MT (1993). Racism, sexism, and social class: implications for studies of health, disease, and well-being. Am J Prev Med.

[130] Loutfy M, Tharao W, Logie C, Aden MA, Chambers LA, Wu W (2015). Systematic review of stigma reducing interventions for African/Black diasporic women. J Int AIDS Soc.

[131] Logie C, James L, Tharao W, Loutfy M (2013). Associations between HIV-related stigma, racial discrimination, gender discrimination, and depression among HIV-positive African, Caribbean, and Black women in Ontario, Canada. AIDS Patient Care STDS..

[132] Doyal L (2009). Challenges in researching life with HIV/AIDS: an intersectional analysis of black African migrants in London. Cult Health Sex.

[133] UNAIDS (2007). Reducing HIV stigma and discrimination: A critical part of national AIDS programmes.

[134] Herek GM, Capitanio JP, Widaman KF (2002). HIV-related stigma and knowledge in the United States: prevalence and trends, 1991–1999. Am J Public Health.

[135] Ogden J, Nyblade L (2005). Common at its core: HIV-related stigma across contexts.

[136] UNAIDS (2010). Report presented at the 26th Meeting of the UNAIDS programme coordinating board: Non-discrimination in HIV responses.

[137] Li LP, Wu ZP, Liang L-JP, Lin CP, Guan JMD, Jia MMD (2013). Reducing HIV-Related Stigma in Health Care Settings: A Randomized Controlled Trial in China. Am J Public Health.

