# Supplementary material for: Stigma, HIV and health: a qualitative synthesis
Source: BMC Public Health. 2015 Sep 3;15:848. doi: 10.1186/s12889-015-2197-0 (PMC4557823; doi:10.1186/s12889-015-2197-0)
Supplement: Additional file 1: — Search strategies used for stigma reviews. This file details the electronic and manual search strategies conducted for this review. (PDF 23 kb) [file 12889_2015_2197_MOESM1_ESM.pdf]

Additional Files

Additional File 1 - Search strategies used for stigma reviews

Description: This file details the electronic and manual search strategies conducted for this review.

For the synthesis of qualititative and quantative literature on stigma, HIV and health (stigma systematic review), two search strategies were conducted: 1) electronic database searches; 2) manual searches of literature that included reviewing reference lists of reviews on the topic of stigma; soliciting literature recommendations from content experts; and reviewing reference lists of literature included at the full-paper review stage.

Electronic Database Search

A librarian conducted electronic searches on six databases: CINAHL, Embase, MEDLINE, PsycINFO, Sociological Abstracts, and WHOLIS. For each database, subject headings and/or keyword terms related to HIV/AIDS (e.g., human immunodeficiency, acquired immunodeficiency syndrome), and stigma/discrimination (e.g., stigma*, ostracism, prejudice, stereotyping, discrimination) were used to extract literature, using a date range from January 1996 to current date of search.

Whenever possible, keywords related to quantitative or qualitative methodology were used to limit the search (e.g., EMBASE, MEDLINE). We chose to restrict literature to post-1995 since the introduction of combination antiretrovirals (cART) is often cited in the literature as a key turning point in the transformation of the HIV epidemic. To avoid the exclusion of relevant health-related literature at this stage, the search strategy used key terms for HIV and stigma, then

incorporated health-related inclusion criteria in the review stages. In total, we yielded *8,397* references from the electronic database search.

Databases included in the electronic database search

CINAHL: The Cumulative Index to Nursing and Allied Health Literature (CINAHL) contains references to health literature, predominantly in the areas of nursing, allied health sciences, and biomedicine.

Embase: The Excerpta Medica database (Embase) contains references to biomedical and pharmacological bibliographic literature with a broad national and international scope.

MEDLINE: MEDLARS Online (MEDLINE) contains references to life sciences literature with a concentration in biomedicine.

PsycINFO: The Psychological Information Database (PsycINFO) contains references to psychology and related disciplines including medicine, psychiatry, nursing, and sociology.

Sociological Abstracts: Sociological Abstracts contains references to international literature in sociology and related disciplines in the social and behavioural sciences.

WHOLIS: The World Health Organization library database (WHOLIS) indexes all WHO publications, journals, and technical documents.

Subject headings and key words used in electronic search strategies

Limit Dates: From 1996 to current date

DATABASE	HIV/AIDS	Stigma
CINAHL	**Subject Headings**<br>"HIV+"<br>AIDS serodiagnosis<br>"HIV-Infected Patients+"<br>"HIV Infections+"<br><br>**Key Words:**<br>HIV<br>AIDS<br>human immunodeficiency<br>acquired immunodeficiency syndrome<br>human immunodeficiency<br>acquired immune deficiency syndrome	**Subject Headings**<br>stereotyping<br>social conformity<br>social attitudes<br>"Social isolation+"<br>"Discrimination+"<br>"Negotiation"<br>Stigma<br>Prejudice<br>Shame<br>Fear<br>Self Concept<br>confidence<br><br>**Key Words:**<br>stigma*<br>social discrimination<br>prejudice*<br>social* exclu*<br>social* isolat*<br>ostraci*<br>marginali*<br>social* alienat*<br>social acceptance

DATABASE	HIV/AIDS	Stigma
PsycINFO	**Subject Headings**<br>aids<br>hiv testing<br>exp "aids (attitudes toward)"<br>aids prevention<br>exp aids dementia complex<br>exp hiv<br>exp hiv testing<br><br>**Key Words:**<br>HIV<br>human immunodeficiency<br>acquired immunodeficiency syndrome<br>acquired immune deficiency syndrome<br>AIDS	**Subject Headings**<br>stereotyped attitudes<br>social approval<br>social acceptance<br>exp social discrimination<br>exp prejudice<br>*labeling<br>Attitudes<br>social perception<br><br>**Key Words:**<br>ostrac$<br>stigma$<br>marginal$<br>social discrimination<br>prejudice$<br>social$ exclu$<br>social$ isolate$<br>social$ alienat$<br>social acceptance
MEDLINE	**Subject Headings**<br>exp hiv infections<br>AIDS Serodiagnosis<br>Acquired Immunodeficiency Syndrome<br>HIV<br>HIV long term survivors<br>exp anti-hiv agents<br>exp hiv protease inhibitors<br>exp reverse transcriptase inhibitors<br><br>**Key Words:**<br>HIV<br>human immunodeficiency<br>acquired immunodeficiency syndrome<br>acquired immune deficiency syndrome<br>AIDS	**Subject Headings**<br>Prejudice<br>Shame<br>Self Concept<br>exp social isolation<br>*stereotyping<br>Taboo<br>Vulnerable Populations<br>Social Conformity<br>social perception<br><br>**Key Words:**<br>ostrac$<br>stigma$<br>marginal$<br>social discrimination<br>prejudice$<br>social$ exclu$<br>social$ isolate$<br>social$ alienat$<br>social acceptance

DATABASE	HIV/AIDS	Stigma
EMBASE	**Subject Headings**<br>human immunodeficiency virus antibody<br>exp human immunodeficiency virus<br>hiv associated lipodystrophy<br>human immunodeficiency virus prevalence<br>exp acquired immune deficiency syndrome<br>*wasting syndrome<br>"attitude to aids"<br><br>**Key Words:**<br>HIV<br>human immunodeficiency<br>acquired<br>immunodeficiency syndrome<br>acquired immune deficiency syndrome<br>AIDS	**Subject Headings**<br>stigma<br>shame<br>taboo<br>Self Concept<br>self esteem<br>social isolation<br>social attitude<br><br>**Key Words:**<br>ostrac$<br>stigma$<br>marginal$<br>social discrimination<br>prejudice$<br>social$ exclu$<br>social$ isolate$<br>social$ alienat$<br>social acceptance
Sociological Abstracts	**Key Words:**<br>HIV<br>human immunodeficiency<br>acquired immunodeficiency syndrome<br>acquired immune deficiency syndrome<br>AIDS	**Key Words:**<br>stigma<br>discriminate<br>discrimination<br>prejudice<br>marginalize<br>marginalization<br>social isolation
WHOLIS	**Key Words:**<br>HIV<br>AIDS	**Key Words:**<br>stigma<br>discrimination<br>prejudice<br>marginalization<br>social isolation

Example of Search Strategy: PsycINFO

Database: PsycINFO <1987 to February Week 3 2010> Search Strategy:

----

1 aids hiv testing exp "aids (attitudes
toward)" aids prevention exp aids dementia complex exp hiv exp hiv testing/ (22467)
2 (hiv or human immunodeficiency or acquired
immunodeficiency syndrome or acquired immune deficiency syndrome or aids).tw. (30560)
3 stereotyped attitudes/ (5468)
4 social approval social acceptance exp social
discrimination *labeling attitudes social perception/ (26136)
5 exp prejudice/ (2676)
6 (ostrac$ or stigma$ or marginal$ or social
discrimination or prejudice$ or social$ exclu$ or social$ isolate$).tw. or social$ alienat$.mp. or social acceptance.tw. (23666)
7 or/1-2 (30680)
8 or/3-6 (51608)
9 7 and 8 (2262)
10 limit 9 to yr=1997-2009 (1677)
11 from 10 keep 1-1000 (1000)
12 from 10 keep 1001-1677 (677)
13 from 10 keep 1 (1)

PsycINFO total = 1,677

Manual Searches

Manual searches were also conducted to better ensure that stigma-related literature not found using the database search was included in the review. Manual search strategies include:

- References identified in other systematic reviews on the topic of stigma;
- References recommended by content experts;
- References identified through manual search of included literature

Literature identified from previously published systematic reviews on stigma

In spring 2009, the review team conducted a citation search of systematic reviews addressing the topic of HIV-related stigma for people living with HIV/AIDS (PHAs). Six reviews were identified (Brown, Macintyre, & Trujillo, 2003; Crawford, 1996; Gadalla & Logie, 2009; Mak, Poon, Pun, & Cheung, 2007; Sandelowski, Lambe, & Barroso, 2004; Vidanapathirana, Randeniya, & Operario, 2007. From the manual review of other systematic reviews, 111 references were identified as being relevant to this review.

Literature identified by content experts

The review team also contacted and received references from content experts with expertise in the literature related to HIV stigma and discrimination. From the papers recommended by content experts, an additional 27 papers were added that were not already included in the review.

Literature identified through manual search

Over the course of the review process, the review team conducted manual searches of references from articles included at Level 2 – full paper review. 87 references were identified from the manual search.

Search yields from electronic and manual searches

Source	# of references
Electronic Database Search	
CINAHL	2,607
EMBASE	1,490
MEDLINE	1,835
PsycINFO	1,677
Sociological Abstracts	766
WHOLIS	22
Manual Searches	
Citation search of systematic reviews	111
Content experts recommendation	27
Manual search of references	87
**Total References**	**8,622**
Duplicates	(2,893)
**Total references included for T&A review**	**5,729**
