# Supplementary material for: Stigma, HIV and health: a qualitative synthesis
Source: BMC Public Health. 2015 Sep 3;15:848. doi: 10.1186/s12889-015-2197-0 (PMC4557823; doi:10.1186/s12889-015-2197-0)
Supplement: Additional file 2: — Articles excluded from qualitative data extraction. This file provides a reference list of papers excluded from the qualitative synthesis at data extraction (i.e. studies that reported only quantitative findings, and studies published in other languages than English, French or Spanish). (PDF 24 kb) [file 12889_2015_2197_MOESM2_ESM.pdf]

Additional Files

Additional file 2 - Articles excluded from qualitative data extraction

Description: This file provides a reference list of papers excluded from the qualitative synthesis at data extraction (i.e. studies that reported only quantitative findings, and studies published in other languages than English, French or Spanish

Articles excluded due to method (reported only quantitative data) (n=65)

1. Berger BE, Ferrans CE, Lashley FR. Measuring stigma in people with HIV: Psychometric assessment of the HIV Stigma Scale. *Res. Nurs. Health.* 2001;24(6):518-529.

2. Bozarth MS. *The relationship of the perception of stigma, depression, social support, and spirituality to the health status of the individual with HIV/AIDS* [Doctorate of Philosophy]. Tallahassee, FL: Department of Human Services and Studies, The Florida State University; 1998.

3. Brennan CS. *Characteristics that affect a HIV infected individual's utilization of HIV medical care* [Doctorate of Philosophy]. Hattiesburg, MS: Nursing, University of Southern Mississippi; 2007.

4. Buseh AG, Kelber ST, Hewitt JB, Stevens PE, Park CG. Perceived stigma and life satisfaction: Experiences of urban African American men living with HIV/AIDS. *Int J Mens Health.* 2006;5(1):35-51.

5. Buseh AG, Kelber ST, Stevens PE, Park CG. Relationship of symptoms, perceived health, and stigma with quality of life among urban HIV-infected African American men. *Public Health Nurs.* 2008;25(5):409-419.

6. Carlucci JG, Kamanga A, Sheneberger R, et al. Predictors of adherence to antiretroviral therapy in rural Zambia. *J Acquir Immune Defic Syndr.* 2008;47(5):615-622.

7. Charney ME. *Understanding the psychological, psychosocial, and physical health status of HIV-positive refugees* [Doctorate of Philosophy]. Boston, MA: Psychology, Boston University; 2008.

8. Clark HJ, Lindner G, Armistead L, Austin B. Stigma, disclosure, and psychological functioning among HIV-infected and non-infected African-American women. *Women Health.* 2003;38(4):57-71.

9. Courtenay-Quirk C, Wolitski RJ, Parsons JT, mez CA. Is HIV/AIDS stigma dividing the gay community? Perceptions of HIV-positive men who have sex with men. *AIDS Educ. Prev.* 2006;18(1):56-67.

10. Demi A, Bakeman R, Sowell R, Moneyham L, Seals B. Suicidal thoughts of women with HIV infection: Effect of stressors and moderating effects of family cohesion. *J. Fam. Psychol.* 1998;12(3):344-353.

11. DiIorio C, McCarty F, DePadilla L, et al. Adherence to antiretroviral medication regimens: A test of a psychosocial model. *AIDS Behav.* 2009;13(1):10-22.

12. Dlamini PS, Wantland D, Makoae LN, et al. HIV stigma and missed medications in HIV-positive people in five African countries. *AIDS Patient Care STDS.* 2009;23(5):377-387.

13. Freeman M, Nkomo N, Kafaar Z, Kelly K. Factors associated with prevalence of mental disorder in people living with HIV/AIDS in South Africa. *AIDS Care.* 2007;19(10):1201-1209.

14. Heckman TG. The chronic illness quality of life (CIQOL) model: explaining life satisfaction in people living with HIV disease. *Health Psychol.* 2003;22(2):140-147.

15. Heckman TG, Anderson ES, Sikkema KJ, Kochman A, Kalichman SC, Anderson T. Emotional distress in nonmetropolitan persons living with HIV disease enrolled in a telephone-delivered, coping improvement group intervention. *Health Psychol.* 2004;23(1):94-100.

16. Heckman TG, Heckman BD, Kochman A, Sikkema KJ, Suhr J, Goodkin K. Psychological symptoms among persons 50 years of age and older living with HIV disease. *Aging Ment. Health.* 2002;6(2):121-128.

17. Heckman TG, Kochman A, Sikkema KJ. Depressive symptoms in older adults living with HIV disease: Application of the Chronic Illness Quality of Life Model. *J Ment Health Aging.* 2002;8(4):267-279.

18. Kalichman SC, Simbayi LC, Cloete A, Mthembu PP, Mkhonta RN, Ginindza T. Measuring AIDS stigmas in people living with HIV/AIDS: The Internalized AIDS-Related Stigma Scale. *AIDS Care.* 2009;21(1):87-93.

19. Kang E, Rapkin BD, DeAlmeida C. Are psychological consequences of stigma enduring or transitory? A longitudinal study of HIV stigma and distress among Asians and Pacific Islanders living with HIV illness. *AIDS Patient Care STDS.* 2006;20(10):712-723.

20. Kang E, Rapkin BD, Remien RH, Mellins CA, Oh A. Multiple dimensions of HIV stigma and psychological distress among Asians and Pacific Islanders living with HIV illness. *AIDS Behav.* 2005;9(2):145-154.

21. Katz S, Nevid JS. Risk factors associated with posttraumatic stress disorder symptomatology in HIV-infected women. *AIDS Patient Care STDS.* 2005;19(2):110-120.

22. Keeney EM. *Traumatic events and PTSD symptomatology in HIV-infected adults and the impact on treatment adherence* [Doctorate of Philosophy]. New York, NY: School of Social Work, New York University; 2008.

23. Kinsler JJ, Wong MD, Sayles JN, Davis C, Cunningham WE. The effect of perceived stigma from a health care provider on access to care among a low-income HIV-positive population. *AIDS Patient Care STDS.* 2007;21(8):584-592.

24. Lang SS. *Social work, psychoneuroimmunology, and HIV/AIDS: An examination of the relationship among health-related hardiness, HIV/AIDS locus of control, stigma, culture, and immune function in persons with HIV/AIDS* [Doctorate of Philosophy]. Denver, CO: Social Work, University of Denver; 2001.

25. Lee RS, Kochman A, Sikkema KJ. Internalized stigma among people living with HIV-AIDS. *AIDS Behav.* 2002;6(4):309-319.

26. Li L, Lee SJ, Thammawijaya P, Jiraphongsa C, Rotheram-Borus MJ. Stigma, social support, and depression among people living with HIV in Thailand. *AIDS Care.* 2009;21(8):1007-1013.

27. Li X, Wang H, Williams A, He G. Stigma reported by people living with HIV in south central China. *J Assoc Nurses AIDS Care.* 2009;20(1):22-30.

28. Lyon DE. *A causal model of the effects of existential well-being on the relationship between severity of human immunodeficiency virus (HIV) disease and depression* [Doctorate of Philosophy]. Richmond, VA: Nursing, Virginia Commonwealth University; 1997.

29. Mak WWS, Cheung RYM, Law RW, Woo J, Li PCK, Chung RWY. Examining attribution model of self-stigma on social support and psychological well-being among people with HIV+/AIDS. *Soc. Sci. Med.* 2007;64(8):1549-1559.

30. Miles MS, Burchinal P, Holditch-Davis D, Wasilewski Y. Personal, family, and health-related correlates of depressive symptoms in mothers with HIV. *J. Fam. Psychol.* 1997;11(1):23-34.

31. Miles MS, Gillespie JV, Holditch-Davis D. Physical and mental health in African American mothers with HIV. *J Assoc Nurses AIDS Care.* 2001;12(4):42-50.

32. Mogengege MA. *If mama's not happy, nobody's happy: An exploration of the influence of stigma, disclosure and family coping on the transmission of maternal depression to children in African-American families affected by HIV/AIDS* [Doctorate of Philosophy]. Washington, D.C.: Black Studies, Howard University; 2008.

33. Moneyham L, Seals B, Sowell R, Hennessy M, Demi A, Brake S. The impact of HIV on emotional distress of infected women: cognitive appraisal and coping as mediators. *Sch. Inq. Nurs. Pract.* 1997;11(2):125-151.

34. Murphy DA, Austin EL, Greenwell L. Correlates of HIV-related stigma among HIV-positive mothers and their uninfected adolescent children. *Women Health.* 2006;44(3):19-42.

35. Naar-King S, Bradford J, Coleman S, Green-Jones M, Cabral H, Tobias C. Retention in care of persons newly diagnosed with HIV: Outcomes of the Outreach Initiative. *AIDS Patient Care STDS.* 2007;21:Supplement.

36. Nachega JB, Stein DM, Lehman DA, et al. Adherence to antiretroviral therapy in HIV-infected adults in Soweto, South Africa. *AIDS Res. Hum. Retroviruses.* 2004;20(10):1053-1056.

37. Pakdewong P, Kasemkitwatana S, Puncharoenworakul K, Miles MS, Kaemkate W. A structural model of maternal role attainment in Thai HIV sero-positive mothers. *Thai J Nurs Res.* 2006;10(3):201-214.

38. Peretti-Watel P, Spire B, Pierret J, Lert F, Obadia Y. Management of HIV-related stigma and adherence to HAART: Evidence from a large representative sample of outpatients attending French hospitals (ANRS-EN12-VESPA 2003). *AIDS Care.* 2006;18(3):254-261.

39. Prachakul W. *Assessment of social support, HIV-related stigma, and social problem-solving as model components of depressive symptomatology in persons with HIV* [Doctorate of Philosophy]. Birmingham, AL: Nursing, University of Alabama at Birmingham; 2005.

40. Prachakul W, Grant JS, Keltner NL. Relationships among functional social support, HIV-related stigma, social problem solving, and depressive symptoms in people living with HIV: A pilot study. *J Assoc Nurses AIDS Care.* 2007;18(6):67-76.

41. Preau M, Bouhnik A, Peretti-Watel P, Obadia Y, Spire B. Suicide attempts among people living with HIV in France. *AIDS Care.* 2008;20(8):917-924.

42. Rajabiun S, Rumptz MH, Felizzola J, et al. The impact of acculturation on Latinos' perceived barriers to HIV primary care. *Ethn. Dis.* 2008;18(4):403-408.

43. Reece M. HIV-related mental health care: Factors influencing dropout among low-income, HIV positive individuals. *AIDS Care.* 2003;15:707-716.

44. Reece MD. *Dropout from community-based, HIV-related psychotherapy: The influence of health beliefs and perceptions of stigma (immune deficiency)* [Doctorate of Philosophy]. Athens, GA, University of Georgia; 2001.

45. Relf MV, Mallinson K, Pawlowski L, Dolan K, Dekker D. HIV-related stigma among persons attending an urban HIV clinic. *J Multicult Nurs Health.* 2005;11(1):14-22.

46. Riggs SA, Vosvick M, Stallings S. Attachment style, stigma and psychological distress among HIV+ adults. *J Health Psychol.* 2007;12(6):922-936.

47. Rintamaki LS, Davis TC, Skripkauskas S, Bennett CL, Wolf MS. Social stigma concerns and HIV medication adherence. *AIDS Patient Care STDS.* 2006;20(5):359-368.

48. Rosenblum Katz S. *Risk factors of post-traumatic stress disorder symptomatology in HIV-infected women* [Doctorate of Philosophy]. New York City, NY: Psychology, St. John's University; 2004.

49. Ryan K, Forehand R, Solomon S, Miller C. Depressive symptoms as a link between barriers to care and sexual risk behavior of HIV-infected individuals living in non-urban areas. *AIDS Care.* 2008;20(3):331-336.

50. Sayles JN, Hays RD, Sarkisian CA, Mahajan AP, Spritzer KL, Cunningham WE. Development and psychometric assessment of a multidimensional measure of internalized HIV stigma in a sample of HIV-positive adults. *AIDS Behav.* 2008;12(5):748-758.

51. Schuster MA, Collins R, Cunningham WE, et al. Perceived discrimination in clinical care in a nationally representative sample of HIV-infected adults receiving health care. *J. Gen. Intern. Med.* 2005(9):807-813.

52. Silver EJ, Bauman LJ, Camacho S, Hudis J. Factors associated with psychological distress in urban mothers with late-stage HIV/AIDS. *AIDS Behav.* 2003;7(4):421-431.

53. Simbayi LC, Kalichman S, Strebel A, Cloete A, Henda N, Mqeketo A. Internalized stigma, discrimination, and depression among men and women living with HIV/AIDS in Cape Town, South Africa. *Soc. Sci. Med.* 2007;64(9):1823-1831.

54. Sohler N, Li X, Cunningham C. Perceived discrimination among severely disadvantaged people with HIV infection. *Public Health Rep.* 2007;122(3):347-355.

55. Sowell RL, Seals BF, Moneyham L, Demi A, Cohen L, Brake S. Quality of life in HIV-infected women in the south-eastern United States. *AIDS Care.* 1997;9(5):501-512.

56. Stutterheim SE, Pryor JB, Bos AER, Hoogendijk R, Muris P, Schaalma HP. HIV-related stigma and psychological distress: The harmful effects of specific stigma manifestations in various social settings. *AIDS.* 2009;23(17):2353-2357.

57. Thomas BE, Rehman F, Suryanarayanan D, et al. How stigmatizing is stigma in the life of people living with HIV: A study on HIV positive individuals from Chennai, South India. *AIDS Care.* 2005;17(7):795-801.

58. Thrasher AD, Earp JA, Golin CE, Zimmer CR. Discrimination, distrust, and racial/ethnic disparities in antiretroviral therapy adherence among a national sample of HIV-infected patients. *J Acquir Immune Defic Syndr.* 2008;49(1):84-93.

59. Vanable PA, Carey MP, Blair DC, Littlewood RA. Impact of HIV-related stigma on health behaviors and psychological adjustment among HIV-positive men and women. *AIDS Behav.* 2006;10(5):473-482.

60. Vance DE. Self-rated emotional health in adults with and without HIV. *Psychol. Rep.* 2006;98(1):106-108

61. Waite KR, Paasche-Orlow M, Rintamaki LS, Davis TC, Wolf MS. Literacy, social stigma, and HIV medication adherence. *J. Gen. Intern. Med.* 2008;23(9):1367-1372.

62. Wight RG. Precursive depression among HIV infected AIDS caregivers over time. *Soc. Sci. Med.* 2000;51(5):759–770.

63. Wingood GM, DiClemente RJ, Mikhail I, et al. HIV discrimination and the health of women living with HIV. *Women Health.* 2007;46(2-3):99-112.

64. Wright K, Naar-King S, Lam P, Templin T, Frey M. Stigma scale revised: Reliability and validity of a brief measure of stigma for HIV+ youth. *J. Adolesc. Health.* 2007;40(1):96-98.

65. Wu DY, Munoz M, Espiritu B, et al. Burden of depression among impoverished HIV-positive women in Peru. *J Acquir Immune Defic Syndr*. 2008;48(4):500-504.

Articles excluded due to language restrictions (studies published in languages other than French, Spanish or English) (n=11)

66. 1. Beier S. African Migrants in Germany and Their Way of Coping with HIV/AIDS [German]. curare. 2005;2-3( ):188-200.

67. 2. Cao XB, Pang L, Wu ZY. Reasons and patterns of AIDS stigma and intervention strategies [Chinese]. Chinese Journal of AIDS/STD. 2005;11(3):243-245.

68. 3. Ferreira RCM, Figueiredo MAdC. Reinserção no mercado de trabalho. Barreiras e silêncio no enfrentamento da exclusão por pessoas com HIV/AIDS [Replacement in the work market. Barriers and silence in the coping of the exclusion for people with HIV/AIDS]. [Portuguese] Medicina (Ribeirao Preto. Online). 2006;39(4):591-600.

69. 4. Figueiredo MAdC, Fioroni LN. Uma análise de conteúdo de crenças relacionadas com a AIDS entre participantes em O.N.G.s [A content analysis of beliefs related to AIDS among NGOs participants]. [Portuguese]. Estudos de Psicologia. 1997;2(1):doi: 10.1590/S1413-1294X1997000100003

70. 5. Li X, He G, Wang H. Study of stigma and discrimination related to HIV and AIDS [Chinese]. Chinese Journal of Nursing. 2007;42:78-80.

71. 6. Melchior R, Nemes MI, Alencar TM, Buchalla CM. Desafios da adesão ao tratamento de pessoas vivendo com HIV/AIDS no Brasil. [Challenges of treatment adherence by people living with HIV/AIDS in Brazil] [Portuguese]. Rev. Saude Publica. 2007;41 (Suppl 2):87-93.

72. 7. Savin K. Stigmatizacija HIV pozitivnih zavisnika od droga u okviru medicinskog sistema [Stigmatization of HIV-positive drug addicts within the medical system] [Serbian]. Socioloski Pregled. 1996;30(2):193-204.

73. 8. Tunala LG. Daily sources of stress among HIV-positive women. [Portuguese]. Rev. Saude Publica. 2002;36(4 Suppl):24-31.

74. 9. Voseckova A, Pelcak S, Sansevicova E. Psychosocial aspects of testing HIV infection. [Czech]. Prakticky Lekar. 1999;79(11):615-617.

75. 10. Wang Y, Dong H, Zhang Y, Zhang R, Lu L. The mental problems and needs in patients under AIDS/HIV discrimination [Chinese]. Chinese Remedies & Clinics. 2010;7:524-526.

76. 11. Yan J, Xiao Sy, Zhou L, Tang Y. Development of perceived stigma scale for children affected by HIV/AIDS. [Chinese]. Chinese Journal of Clinical Psychology. 2008;16(2):129.
