# Supplementary material for: Stigma, HIV and health: a qualitative synthesis
Source: BMC Public Health. 2015 Sep 3;15:848. doi: 10.1186/s12889-015-2197-0 (PMC4557823; doi:10.1186/s12889-015-2197-0)
Supplement: Additional file 3: — Summary of study characteristics. This file summarizes key characteristics of included studies (i.e., study aims/objectives, geographic jurisdiction, characteristics of study sample, and concept of stigma applied in the study). (PDF 37 kb) [file 12889_2015_2197_MOESM3_ESM.pdf]

Additional Files

Additional File 3 - Summary of study characteristics

Description: This file summarizes key characteristics of included studies

First Author (Year)	Aims/Objectives (Method)	Jurisdiction (Income Class)	Sample Characteristics	Concept of Stigma
Agne (2000)	To describe the major influential factors prompting the act of disclosure of HIV, the act itself, and the effect that act appears to have had on the patient/provider relationship.	United States (High): Midwest, West Coast, South and Northwest part of the United States	107 HIV-positive individuals	Goffman's concept of stigma (1963)
Anderson (2008)	To explore the effects of HIV/AIDS-related stigma and discrimination (HASD) on HIV-positive Caribbean people in the Caribbean and the UK.	United Kingdom (High): South London, UK	25 PLHIV of Caribbean origin	Not explicitly defined
Balabanova (2006)	To examine the personal and social consequences of stigma associated with HIV infection in Russia.	Russian Federation (Upper-Mid): Samara Oblast	64 participants recruited randomly from Samara city population; 45 PLHIV recruited from regional AIDS clinic.	Not explicitly defined
Barnes (2000)	To examine from HIV-positive women's perspectives, how they were told their positive HIV antibody test results, what information they were given, and their reactions to this information.	United States (High): Oakland, California; Chicago, Illinois; Rochester, New York	70 HIV-positive women, recruited by community and social workers, from social service agencies serving people with HIV/AIDS in selected study settings.	Not explicitly defined
Bikaako-Kajura (2006)	To describe contextual factors influencing adherence to daily drug regimens among paediatric and adolescent HIV/AIDS patients; to identify and describe barriers and facilitators of adherence; and to identify relevant social support mechanisms that enhance drug adherence among children.	Uganda (Low): Kampala	42 HIV-infected registered paediatric patients who were on ARTs (ages 5-17); 42 primary caregivers	Not explicitly defined

First Author (Year)	Aims/Objectives (Method)	Jurisdiction (Income Class)	Sample Characteristics	Concept of Stigma
Brickley (2007)	To understand the experience of being an HIV+ pregnant woman in Vietnam and their experience accessing and utilizing health care.	Vietnam (Lower-Mid): Ho Chi Minh City	20 HIV+ women who were pregnant, or who had given birth/terminated birth in the past year; 16 key informants (midwives, doctors who have treated HIV+ pregnant women)	Goffman's concept of stigma(1963)<br>Concept of the stigma trajectory (Alonzo and Reynolds, 1995)<br>Impact of AIDS stigma on the prevention-to care continuum (Busza, 2001)<br>Conceptual Framework of Stigma (Parker and Aggleton, 2003)
Brion (2007)	To describe the medication adherence experiences of HIV positive gay men who self reported being adherent to their medication regimen by describing facilitates of successful medication adherence and challenges to medication adherence.	United States (High): Columbus, Ohio	24 HIV+ gay men	Not explicitly defined
Buseh (2006)	To exchange experiences and ideas about living with HIV and to describe PHLAs' perspective on challenges and opportunities for HIV prevention and care among African American men.	United States (High): Midwest metropolitan area	20 HIV+ African American men	Not explicitly defined
CAAT (2008)	To collect stories and information about the life experiences of IRN-PLHIV and how the stress they face daily affect their mental health.	Canada (High): Greater Toronto Area (GTA), Ontario	47 immigrants, refugees and non-status PHA; 28 service providers(frontline workers, administrators, funders, policy makers) who worked in HIV/AIDS, immigration and settlement, or mental health)	Not explicitly defined
Cain (2001)	To describe self-perceived health-related quality of life among individuals living with HIV disease who resided in a selected rural area of Pennsylvania.	United States (High): Rural area of Pennsylvania	150 HIV+ persons who lived in rural communities	Not explicitly defined

First Author (Year)	Aims/Objectives (Method)	Jurisdiction (Income Class)	Sample Characteristics	Concept of Stigma
Cao (2006)	To identify the reasons, sources, and types of HIV-related stigma prevalent in rural China.	China (Upper-Mid): Fuyang Prefecture, Anhui	20 PLHIV who were former plasma donors (FPDs); 20 immediate family members of PLHIV; 20 local health workers; and 20 uninfected villagers, including eight people who were village leaders	Not explicitly defined
Carr (2004)	To explore the multidimensional effect of stigma on women's efforts to promote, maintain, and enhance their health.	United States (High): Region not specified	9 HIV+ European American women	Goffman's concept of stigma(1963)
Castro (1998)	To examine how people affected directly or indirectly by the HIV/AIDS epidemic cope with HIV-related illness in Mexico by describing the AIDS-related wellness/illness careers or trajectories followed by individuals in an economically and sexually (gay) marginalized community, and to identify critical points for material and emotional intervention.	Mexico (Upper-Mid): Ciudad Netzahualcoyotl and Mexico city	29 PLHIV; 45 relatives of PLHIV; 39 friends of PLHIV	Not explicitly defined
Dawson-Rose (2005)	To examine health care experiences of HIV-positive people who use injecting drugs.	United States (High): New York, NY and San Francisco, CA	161 PLHIV who were currently using injecting drugs	Not explicitly defined
Edwards (2006)	To investigate the women's perceptions of social support and how it affected their medication adherence.	United States (High): Baltimore, Maryland	20 HIV+ African American women	Not explicitly defined
Elamon (2005)	To map the forms of structural discrimination and occurrence of arbitrary discrimination (i.e., unjustified discrimination) that inform the lives of PLWHA in India, with a focus on the area of health care.	India (Lower-Mid): Trivandrum district, in the Indian southern state of Kerala	20 PLHIV; 12 key informants (doctors, nurses, laboratory technicians, HIV counsellors, ward attendants and NGO workers)	UNAIDS protocol for the identification of discrimination against People Living with HIV
Emlet (2007)	To examine the experiences of HIV-related stigma in older adults with HIV/AIDS.	United States (High): Pacific Northwest	25 older PLHIV (50 years or older)	Goffman's concept of stigma (1963)

First Author (Year)	Aims/Objectives (Method)	Jurisdiction (Income Class)	Sample Characteristics	Concept of Stigma
Erwin (1999)	To explore the feelings and beliefs of Black Africans in London, when it comes to HIV treatment and treatment concerns, and discuss how they may affect treatment update of adherence.	United Kingdom (High): London	44 Black African immigrants from Uganda, Zambia, Ethiopia, Nigeria, Kenya, Zimbabwe and Tanzania	Not explicitly defined
Gardezi (2008)	To understand HIV-related stigma, discrimination, denial and fear, and the effects of multiple intersecting factors that influence responses to the disease, prevention practices and access to treatment and support services.	Canada (High): Toronto, ON	30 HIV+ individuals; 74 community members whose HIV status was negative or unknown who were born in or parents born in Guyana, Jamaica Trinidad, Ethiopia, Kenya or Somalia	Not explicitly defined
Gaudine (2007)	To describe HIV-related stigma from the perspective of three groups within one community in Vietnam: people living with HIV, their family members and community members and leaders, including health care professionals.	Vietnam (Lower-Mid): Poor, industrial district on the outskirts of a large city in Vietnam	18 PHA, 18 family members (immediate family and immediate in-laws), 10 community members, 12 community leaders(e.g., teachers, health care providers, or representative of the women's, youth, or labour union).	Concept of stigma emerged from study findings
Geurtsen (2005)	To explore the unique meanings people living with HIV/AIDS in Cambodia attach to quality of life (QOL).	Cambodia (Low): Siem Reap and Phnom Phen	25 HIV-positive participants	Not explicitly defined

First Author (Year)	Aims/Objectives (Method)	Jurisdiction (Income Class)	Sample Characteristics	Concept of Stigma
Greeff (2007)	To explore the meaning of stigma from the perspective of people living with HIV and the nurses involved in their care, and how this has affected people living with HIV.	South Africa (Upper-Mid): North West Province: Potchefstroom (urban) and Kayakulu (rural)	40 PLWA and nurses involved in their care	Goffman's concept of stigma(1963); Concept of the stigma trajectory (Alonzo and Reynolds, 1995)
Green (1997)	To examine how stigma affects the relationship between people with HIV and health care workers, and to identify the mechanisms through which it operates in health care settings in Scotland.	United Kingdom (High): Central belt of Scotland	61 people living with HIV	Not explicitly defined
Herrera (2008) (Span)	To analyze changes and continuities that HIV has brought to the interaction between patients and medical staff.	Mexico (Upper-Mid): States of Guerrero, Morelos and the Federal District of Mexico.	40 people living with HIV / AIDS; five leaders of support groups.	Not explicitly defined
Ingram (1999)	To determine how stigma pervades the lives of HIV-positive mothers and their children.	United States (High): Florida	18 HIV-positive mothers	Goffman's (1963) concept of stigma
Konkle-Parker (2008)	To elicit perceived barriers and facilitators for HIV medication adherence in a primarily African American, low-income sample in Mississippi to support the development of an adherence intervention.	United States (High): Mississippi	20 HIV-infected clients of a large public infectious diseases clinic	Not explicitly defined
Kumarasamy (2005)	To examine barriers and facilitators of ART adherence among patients receiving HIV primary care at YRG CARE, a non-governmental organization, in Chennai, India.	India (Lower-Mid): Chennai, India	60 HIV+ patients receiving HIV primary care at YRG CARE	Not explicitly defined
Lindau (2006)	To elicit HIV+ mothers (1) motivations for childbearing, (2) experiences with the health care and child welfare systems, and (3) perspectives on possible strategies to prevent perinatal HIV transmission.	United States (High): Illinois	15 women who had given birth with knowledge of their HIV seropositivity, to at least two children	Not explicitly defined

First Author (Year)	Aims/Objectives (Method)	Jurisdiction (Income Class)	Sample Characteristics	Concept of Stigma
Maher (2007)	To explore, from the perspective of PLWHA, challenges and opportunities for improving access to HIV treatment, care and support in Vietnam	Vietnam (Lower-Mid): Ho Chi Minh City, Vietnam	38 PLWHAs who use injecting drugs Participant Focus groups were divided into groups for younger IDUs, older IDUs, women and female sex workers (FSWs)	Not explicitly defined
Mill (2003).	To examine the experience of HIV-positive Ghanaian women and to identify factors that influenced their vulnerability to infection.	Ghana (Lower-Mid): Accra, Agomanya	31 HIV+ women	Not explicitly defined
Mills (2006)	To explore the nature and dynamics of HIV-related stigma in the KTC with a view to understand the impact of stigma on the lives of HIV+ people and their social environment.	South Africa (Upper-Mid): KTC, an informal settlement in Cape Town	Five Luvuyolwethu carers and their clients(approximately 40 HIV+ people and 40 people in the fourth stage of the disease) and taking antiretroviral treatment	Goffman's concept of stigma (1963); "symbolic stigma" as defined by Herek and Capitanio (1988) "Courtesy stigma" as defined by Kleinman (1995)
Murray (2009)	To gain local insight into potentially important factors affecting HIV-infected women's decision to accept or continue with ART.	Zambia (Lower-Mid)	47 HIV-infected women; 33 key informants with knowledge of issues around HIV adherence	Not explicitly defined
Muyinda (1997)	To examine the perceptions of AIDS as an illness, manifestations of stigma, and to define health and social problem arising from stigmatization, and how individuals and their families can better cope with these situations.	Uganda (Low): South West Uganda.	127 respondents; of those 24 respondents offered information on stigma related to HIV infection: 9 caregivers of PLHIV; 9 individuals suspected of being HIV-positive in community; 6 PLHIV	Goffman's concept of stigma (1963)
Napravnik (2000)	To understand issues affecting prenatal care access and utilization from the perspectives of HIV+ women who receive inadequate prenatal care.	United States (High): North Carolina	3 HIV+ women who reported receiving inadequate prenatal care based on the Kotelchuck index	Not explicitly defined

First Author (Year)	Aims/Objectives (Method)	Jurisdiction (Income Class)	Sample Characteristics	Concept of Stigma
Nguyen (2008)	To investigate the access of HIV-infected pregnant women to PMTCT services in the well-resourced setting of the capital city, Hanoi, Vietnam; specifically how many women received minimal, comprehensive, and optional PMTCT services; and what were their experiences in accessing PMTCT services in a well-resourced urban setting.	Vietnam (Lower-Mid): Hanoi, Vietnam	52 HIV-infected women who found out that they were HIV positive before or during pregnancy and had completed the pregnancy	Not explicitly defined
Pugatch (2002)	To identify major factors which may correlate with HIV-positive adolescent adherence to drug regimens.	United States (High): Rhode Island, US.	Six adolescent patients (ages 13-24) who were currently taking HIV medication	Not explicitly defined
Rajabiun (2007)	To investigate participants' experiences with HIV medical care from a first-person perspective in order to understand how outreach interventions influence engagement and retention in care.	United States (High): Six cities in the northwest, midwest, mid-Atlantic, and northeast regions of the United States.	76 participants living with HIV/AIDS in six cities, from seven sites that participated in the HRSA-funded Outreach Initiative.	Not explicitly defined
Rao (2007)	To examine the obstacles youth face in adhering to HAART and to identify these barriers and to systematically examine the experiences and attitudes youth have towards medications.	United States (High): Chicago	25 adolescents and young adults presenting to a public facility for treatment of HIV infection	Not explicitly defined
Rintamaki (2007)	To explore patients' reported perceptions of and experiences with HIV stigma in health care contexts.	United States (High): large city in the Midwestern United States	50 male American military veterans living with HIV	Not explicitly defined
Roberson (2007)	To explore incarcerated HIV-infected women's barriers to and facilitators of adherence to antiretroviral therapy (ART), the role of health care provider relationships in adherence, and the ways in which issues of medical privacy influence ability on desire to adhere while incarcerated.	United States (High)	12 HIV-positive female inmates who were prescribed ART either by directly observed therapy (DOT), keep own prescription (KOP), or both.	Not explicitly defined
Roberts (2005)	To examine the adherence patterns of HIV-positive children, using the words and voices of both the children themselves and their guardians.	United States (High): Los Angeles, CA	9 HIV+ children (ages 6-12) and 14 guardians of HIV-positive children	Not explicitly defined

First Author (Year)	Aims/Objectives (Method)	Jurisdiction (Income Class)	Sample Characteristics	Concept of Stigma
Rutledge (2009)	To contextualize the experience of AIDS stigma in health services in Grenada and Trinidad and Tobago.	Grenada (Upper-Mid); Trinidad and Tobago (High): Regions not specified	51 PLHIV, families, and service providers divided in to 8 homogenous groups.	Link and Phelan's (2001) Social cognitive stigma framework
Sabin (2008)	To explore barriers to antiretroviral treatment adherence, amongst Chinese patients, most of whom were former IDUs.	China (Upper-Mid): Old Dali, Yunnan province	36 HIV-positive men and women in Dali, in southwestern China.	Not explicitly defined
Sanjobo (2008)	To explore patients' and health care professionals' views on barriers and facilitators to patients' adherence to ART in Zambia.	Zambia (Lower-Mid): Kitwe and Masaiti districts	Purposive sampling60 HIV patients who were on ART; 12 health professionals (doctors, clinical officers, nurses, home-based care, and pharmacists) who had experience with ART	Not explicitly defined
Sayles (2007)	To identify the most salient domains of stigma and the coping strategies that may be common to a group of diverse, low-income women and men living with HIV in Los Angeles, CA; and to explore the impact of stigma on health and health care among HIV+ persons.	United States (High): Los Angeles, CA	Convenience sampling48 HIV-positive persons	Link and Phelan's (2001) Social cognitive stigma framework
Schilder (2001)	To characterize the relationship between identity and health care experiences (including antiretroviral therapy utilization) among HIV-positive sexual minority males.	Canada (High): Vancouver , BC	Purposive sampling47 HIV positive participants from three minorities: gay men, bisexual men and transgendered persons, gender identifying as female and or living as women	Not explicitly defined
Starks (2008)	To identify barriers to and facilitators of medication adherence with PLHAs in China.	China (Upper-Mid) : Beijing	Purposive sampling29 persons receiving HIV care at Beijing's Ditan Hospital	Conceptual model of stigma and discrimination and medication adherence derived from authors' analysis of findings

First Author (Year)	Aims/Objectives (Method)	Jurisdiction (Income Class)	Sample Characteristics	Concept of Stigma
Steward (2008)	To use a newly developed measures of the stigma to assess enacted and internalized stigma and its association with coping strategies designed to avoid disclosure of one’s HIV serostatus leading to psychological distress and depression.	India (Lower-Mid): Southern India.	Convenience samplingStudy 1 : 16 HIV+ participants were recruited in the general medicine department in a large, urban, private hospital in Southern India	Conceptual framework of stigma, avoidance of disclosure and psychological distress that builds on Hidden Distress Model (Scambler, 1989)
Surlis (2001)	To explore, within an Irish context, HIV-positive patients’ experiences ofhospitalization and particularly their experiences of nursing care.	Ireland (High): Dublin	Convenience sampling 10 former hospitals inpatients	Goffman's concept of stigma (1963)
Thi (2008)	To understand the causes and sequelae of stigma and discrimination against PLHIV and more clearly identify the problems faced by PLHIV in Ho Chi Minh City, Vietnam in order to gain insights on the causes, manifestations and impact of stigma and discrimination.	Vietnam (Lower-Mid): Ho Chi Minh City	Purposive sampling53 people living with HIV; divided into seven groups based on gender, age, and common risk characteristics.	Conceptual framework of stigma and discrimination against HIV-positive people in Ho Chi Minh city based on emerging findings
Tippett (2007)	To examine adherence, disclosure and stigma across various dimensions of the child and caregiver's lives.	Botswana (Upper-Mid): Gaborone	Purposive sampling:30 caregivers of HIV-infected children treated during the study period; additional 50 interviews of caregivers, male partners, and pediatric HIV health-care providers	Goffman's concept of stigma (1963) contextualized for African context
Wang (2008)	To investigate discrimination against PLHAs in health care services and its impacts.	China (Upper-Mid): Gejiu City, Honghe Prefecture, Yunnan	Convenience sampling 7 HIV+ individuals; 33 suspected HIV-positive individuals	UNAIDS protocol for the identification of discrimination against People Living with HIV (2000)
Ware (2006)	To examine the intersection between taking HAART and building a life with HIV/AIDS focusing on experiences of illness, drug use and antiretroviral therapy for HIV/AIDS in HIV+ active illegal drug users.	United States (High): Boston, MA	Purposive sampling52 participants living with AIDS, currently on antiretroviral medication, and reported having used illegal drugs in the last 3 months	Conceptual model of stigma and health derived from authors' analysis of findings

First Author (Year)	Aims/Objectives (Method)	Jurisdiction (Income Class)	Sample Characteristics	Concept of Stigma
Wrubel (2005)	To articulate what is involved in the daily life experience of giving or supervising a child's HIV medication (i.e., adherence practices) in order to clarify what promotes or impedes adherence.	United States (High): San Francisco and Oakland, CA; New York, NY	Convenience sampling71 maternal caregivers of children with HIV (including HIV-positive mothers)	Not explicitly defined
Zukoski (2009)	To contextualize and describe how PLHA in a predominantly rural area with low rates of HIV/AIDS experience and respond to perceived acts of stigmatization and discrimination in their day-to-day lives and in health care setting.	United States (High): rural counties in Oregon	Convenience sampling16 adults living with HIV who reside in a predominantly rural area with low HIV prevalence	Based on literature review of stigma; Herek et al. (1998) conceptual framework cited
