# Supplementary material for: Stigma, HIV and health: a qualitative synthesis
Source: BMC Public Health. 2015 Sep 3;15:848. doi: 10.1186/s12889-015-2197-0 (PMC4557823; doi:10.1186/s12889-015-2197-0)
Supplement: Additional file 4: — Matrix summary of qualitative themes. This file classifies the common themes related to HIV, stigma and health as identified in the included studies. (PDF 81 kb) [file 12889_2015_2197_MOESM4_ESM.pdf]

Additional Files

Additional File 4 - Matrix summary of qualitative themes

Description: This file classifies the common themes related to HIV, stigma and health as identified in the included studies.

		Conceptualizing Stigma						Experiencing Stigma					Managing Stigma	
		**Enacted Stigma**	**Felt Stigma**	**Marginaliz-ation**	**Disclosure**	**Morals and Values**	**Visible Health**	**Stigma + Health Care Access**	**Stigma + Health Care Utilization**	**Stigma + Adherence**	**Stigma + Mental Health**	**Stigma + Physical Health**	**Avoiding Stigma**	**Addressing Stigma**
Agne (2000)	*United States*		Y		Y			Y	Y					Y
Anderson (2008)	*United Kingdom*	Y	Y	Y	Y	Y	Y	Y	Y		Y		Y	Y
Balabanova (2006)	*Russian Federation*	Y	Y		Y	Y		Y			Y			Y
Barnes (2000)	*United States*		Y	Y	Y			Y	Y		Y		Y	Y
Bikaako-Kajura (2006)	*Uganda*				Y					Y				Y
Brickley (2007)	*Vietnam*	Y	Y	Y	Y	Y	Y	Y	Y		Y		Y	Y
Brion (2007)	*United States*		Y		Y		Y			Y			Y	
Buseh (2006)	*United States*	Y	Y	Y	Y	Y	Y	Y	Y	Y	Y		Y	Y
CAAT(2008)	*Canada*	Y	Y	Y	Y			Y	Y		Y		Y	Y
Cain (2001)	*United States*		Y	Y				Y	Y				Y	Y
Cao (2006)	*China*	Y	Y	Y	Y	Y		Y	Y				Y	Y
Carr (2004)	*United States*	Y	Y		Y	Y		Y		Y	Y		Y	Y
Castro (1998)	*Mexico*	Y	Y	Y	Y	Y		Y	Y		Y			Y

		Conceptualizing Stigma						Experiencing Stigma					Managing Stigma	
		**Enacted Stigma**	**Felt Stigma**	**Marginaliz-ation**	**Disclosure**	**Morals and Values**	**Visible Health**	**Stigma + Health Care Access**	**Stigma + Health Care Utilization**	**Stigma + Adherence**	**Stigma + Mental Health**	**Stigma + Physical Health**	**Avoiding Stigma**	**Addressing Stigma**
Dawson-Rose (2005)	*United States*	Y	Y	Y	Y	Y		Y	Y				Y	Y
Edwards (2006)	*United States*	Y	Y		Y					Y				Y
Elamon (2005)	*India*	Y	Y		Y			Y						
Emlet (2007)	*United States*	Y	Y	Y	Y	Y		Y						
Erwin (1999)	*United Kingdom*			Y	Y			Y	Y	Y			Y	
Gardezi (2008)	*Canada*	Y		Y	Y	Y		Y	Y					Y
Gaudine (2007)	*Vietnam*	Y	Y		Y	Y		Y					Y	Y
Geurtsen (2005)	*Cambodia*	Y	Y		Y	Y					Y		Y	Y
Greeff (2007)	*South Africa*	Y	Y		Y	Y		Y			Y		Y	
Green (1997)	*United Kingdom*		Y	Y	Y			Y	Y					Y
Herrera (2008)	*Mexico*		Y	Y				Y	Y		Y		Y	
Ingram (1999)	*United States*	Y	Y	Y	Y	Y	Y	Y		Y			Y	Y
Konkle-Parker (2008)	*United States*	Y	Y							Y				Y
Kumarasamy (2005)	*India*	Y	Y		Y								Y	Y
Lindau (2006)	*United States*		Y		Y	Y		Y	Y		Y		Y	Y
Maher (2007)	*Vietnam*	Y		Y	Y	Y		Y	Y					Y
Mill (2003)	*Ghana*	Y	Y	Y	Y	Y	Y		Y		Y			Y
Mills (2006)	*South Africa*	Y		Y	Y	Y		Y	Y					Y
Murray (2009)	*Zambia*		Y	Y						Y	Y			

		Conceptualizing Stigma						Experiencing Stigma					Managing Stigma	
		**Enacted Stigma**	**Felt Stigma**	**Marginaliz-ation**	**Disclosure**	**Morals and Values**	**Visible Health**	**Stigma + Health Care Access**	**Stigma + Health Care Utilization**	**Stigma + Adherence**	**Stigma + Mental Health**	**Stigma + Physical Health**	**Avoiding Stigma**	**Addressing Stigma**
Muyinda (1997)	*Uganda*	Y	Y	Y		Y	Y	Y	Y		Y		Y	Y
Napravnik (2000)	*United States*		Y	Y	Y	Y		Y	Y		Y			Y
Nguyen (2008)	*Vietnam*			Y				Y	Y				Y	Y
Pugatch (2002)	*United States*		Y		Y					Y			Y	Y
Rajabiun (2007)	*United States*		Y	Y				Y	Y		Y		Y	Y
Rao (2007)	*United States*	Y			Y					Y				
Rintamaki (2007)	*United States*	Y				Y		Y						
Roberson (2007)	*United States*	Y	Y	Y	Y			Y		Y				Y
Roberts (2005)	*United States*		Y		Y					Y				
Rutledge (2009)	*Grenada; Trinidad and Tobago*	Y	Y	Y		Y		Y	Y					Y
Sabin (2008)	*China*	Y	Y	Y	Y	Y		Y		Y	Y			Y
Sanjobo (2008)	*Zambia*	Y	Y		Y			Y		Y	Y			Y
Sayles (2007)	*United States*	Y	Y	Y	Y	Y	Y	Y	Y	Y	Y		Y	Y
Schilder (2001)	*Canada*			Y		Y		Y	Y					Y
Starks (2008)	*China*	Y	Y		Y			Y	Y	Y				
Steward (2008)	*India*	Y	Y		Y			Y	Y		Y		Y	
Surlis (2001)	*Ireland*		Y	Y		Y		Y						Y
Thi (2008)	*Vietnam*	Y	Y	Y	Y	Y		Y	Y		Y		Y	Y
Tippett Barr (2007)	*Botswana*	Y	Y	Y	Y	Y	Y			Y				Y

		Conceptualizing Stigma						Experiencing Stigma					Managing Stigma	
		**Enacted Stigma**	**Felt Stigma**	**Marginaliz-ation**	**Disclosure**	**Morals and Values**	**Visible Health**	**Stigma + Health Care Access**	**Stigma + Health Care Utilization**	**Stigma + Adherence**	**Stigma + Mental Health**	**Stigma + Physical Health**	**Avoiding Stigma**	**Addressing Stigma**
Wang (2008)	*China*	Y	Y	Y				Y	Y				Y	Y
Ware (2006)	*United States*	Y	Y	Y	Y				Y	Y	Y		Y	Y
Wrubel (2005)	*United States*		Y		Y					Y				
Zukoski (2009)	*United States*	Y	Y	Y	Y	Y		Y						Y
		**37**	**46**	**34**	**43**	**29**	**9**	**41**	**30**	**20**	**23**	**0**	**27**	**42**
