# Supplementary material for: Stigma, HIV and health: a qualitative synthesis
Source: BMC Public Health. 2015 Sep 3;15:848. doi: 10.1186/s12889-015-2197-0 (PMC4557823; doi:10.1186/s12889-015-2197-0)
Supplement: Additional file 5: — Descriptive summary of themes. This files defines the common themes identified in the qualitative literature related to HIV, stigma and health. (PDF 17 kb) [file 12889_2015_2197_MOESM5_ESM.pdf]

Additional Files

Additional File 5 - Descriptive summary of themes

Description: This files defines the common themes identified in the qualitative literature related to HIV, stigma and health.

CONCEPTUALIZING STIGMA
**Enacted Stigma:** The beliefs, attitudes, behaviours, and individual actions others may hold, demonstrate or enacted against HIV, AIDS and people presumed or living with HIV (i.e., perceptions surrounding HIV and AIDS, emotions elicited when encountering those with HIV, modes of separation used to distance oneself from those who are HIV positive, and forms of discrimination). The structural, organizational or institutional practices that instill perceptions of HIV, AIDS and people presumed or living with HIV.
**Felt Stigma:** The beliefs or attitudes, behaviours and actions people with HIV may hold or demonstrate in response to or in acceptance of devaluing messages around their seropositivity (i.e., emotions expressed when encountering stigma, modes of social and emotional distancing to separate oneself from stigma, valuations of one's sense of self and self-identity once their status is known, identification with discriminatory attitudes, behaviours or actions).
**Marginalization:** Social identities (sexual identity, gender identity, ethnoracial identity, socioeconomic class, age) or activities (drug use, sex work, incarceration history) and their devaluation as they interconnect with HIV; the intersecting or differential experiences of stigma for people with HIV that are shaped by other forms of marginalization .
**Disclosure:** The concerns individuals may have when considering disclosing their status: Should they disclosure? When should they disclose? To whom should they disclose? What should they disclose? What are the consequences of disclosure?
**Morals and Values:** The interconnections between social mores, societal values and stigma such as moralized understandings of HIV-transmitting activities, socially permitted roles of those who are HIV-positive (e.g., role as a intimate sexual partner, role as a mother), and social roles potentially unfilled due to HIV illness (e.g., role as economic provider).
**Visible Health:** Symptoms of HIV illness as a marker of seropositivity (inadvertent disclosure) and as a form of stigma.

EXPERIENCING STIGMA

Stigma & Health Care Access: Experiences of externalized stigma within health care settings such as prejudicial beliefs, attitudes, and behaviours expressed by health care status (e.g., stigmatizing emotional reactions, judgemental attitudes), inadequate care practices (e.g., , substandard care, denied care) and discriminatory practices and procedures (e.g., excessive precautions, segregation of HIV-positive patients, confidentiality violations etc.).

Stigma & Health Care Utilization: Experiences of internalized stigma when utilizing care (e.g., fear of disclosure and selective health care practices, fear of disclosure and non-disclosure to health care practitioners, denial of status and avoidance of care).

Stigma & Adherence: Experiences of stigma when adhering to antiretroviral medications (ARTs) (e.g., antiretrovirals and a signifier of HIV-status, inadvertent disclosure via ART adherence, adherence strategies as a means of mitigating stigma).

Stigma & Mental Health: Experiences of stigma and mental health (e.g., internalized stigma as a contributor to depression, suicide ideation as a means of circumventing stigma, substance use as a means of coping with stigma, anxiety associated with fears of disclosure and subsequent stigma).

MANAGING STIGMA

Avoiding Stigma: Strategies people with HIV use to circumvent or avoid stigma (e.g., deceiving others about their health, self isolation to avoid disclosure, avoiding services or programs associated with HIV, distancing oneself from identities or activities associated with HIV, and avoiding acceptance of status).

Addressing Stigma: Strategies used individually or collectively to address, confront or resist stigma (i.e., emotional or instrumental support, increasing one’s knowledge of HIV, self-advocacy, raising awareness, educating others and social advocacy etc.)
